# Physiological and transcriptomic analysis of cranberry (*Vaccinium macrocarpon*) in response to drought stress

**DOI:** 10.3389/fpls.2026.1797317

**Published:** 2026-05-07

**Authors:** Fanfan Chen, Lei Yang, Haikun Tang, Jiaping Yu, Jinyan Huang, Haiyue Sun, Li Chen, Yadong Li

**Affiliations:** 1 College of Horticulture, Jilin Agricultural University, Changchun, Jilin, China; 2 Jilin Province Engineering Center for Genetic Breeding and Innovative Utilization of Small Fruits, Changchun, Jilin, China

**Keywords:** anatomical structure, cranberry, drought stress, photosystem, transcriptome

## Abstract

Cranberry is a small fruit crop with great development potential. Due to its shallow root system and dominance of fibrous roots, it is highly susceptible to drought stress, but the response mechanism is still unclear. In this study, “Bain 11” cranberry was used as the material, and four pot treatments were set up: normal water supply (CK), mild (D1), moderate (D2), and severe (D3) drought. After reaching the set stress levels, functional leaves were collected. The response mechanism to different degrees of drought was clarified by combining paraffin sectioning, chlorophyll fluorescence, spectrophotometry, and transcriptome sequencing technology. The results showed that there was no significant change in the rate of photosynthetic oxygen release under D1 conditions; under D2 conditions, the function of PSII was impaired, but the heat dissipation capacity was enhanced, and the protective mechanism was activated; under D3 conditions, the performance of both PSII and PSI was significantly inhibited, the damage to the oxygen-evolving complex was the most severe, and the photosynthetic oxygen production decreased significantly. Leaf anatomical structure showed that with the intensification of drought, the morphology, area, and density of stomata changed, and the thickness of palisade tissue, epidermis, and spongy tissue decreased; under D2 conditions, organelle membranes were deformed but intact, and the thylakoid lamellar spacing increased; under D3 conditions, organelle membranes ruptured, nuclear material leaked, chloroplasts vacuolated, and thylakoid lamellae disintegrated. The oxidative stress indicators MDA and H_2_O_2_ increased with the intensity of the stress, and the activities of SOD and CAT significantly increased under D2 and D3 conditions. Transcriptome sequencing identified 58,020 single genes, and KEGG enrichment analysis showed that 55 continuously differentially expressed genes were involved in 54 metabolic pathways (19 upregulated and 36 downregulated), among which photosynthesis-related genes accounted for the highest proportion (13%) and played a key role in the drought response, providing important insights into the drought stress response mechanism of cranberry.

## 1 Introduction

In recent years, attributable to global warming, arid areas in most countries across the globe have been on the rise. Moreover, the scarcity of freshwater resources has emerged as one of the crucial factors influencing crop yields (Lehner et al., 2006; Lesk et al., 2016; Fullana-Pericàs et al., 2018). To achieve efficient utilization of water resources, numerous scholars have focused their research on the effects of drought on plants and the mechanisms by which plants adapt to drought stress. Among various plant metabolic activities, photosynthesis is a metabolic process that is highly sensitive to drought (Sharma and Zheng, 2019). Under drought stress, the decline in photosynthetic rate is mainly attributed to stomatal and non-stomatal limitations. Stomatal limitation results from the closure of leaf stomata. This closure reduces the intercellular CO_2_ concentration, thereby causing a decline in the photosynthetic rate. Non-stomatal limitation results from the impaired functionality of the photosynthetic machinery and the decreased activity of photosynthetic enzymes, which are induced by water deficiency (Varone et al., 2012; Ghotbi‐Ravandi et al., 2014). Under these conditions, the electron transfer equilibrium between light absorption and light utilization within the plant photosystem is disrupted. This disruption leads to the accumulation of excessive excitation energy and the generation of ROS The excessive buildup of ROS triggers lipid peroxidation of cell membranes. This process impedes signal transduction and substance synthesis among cells, ultimately leading to cell death (Jiang et al., 2006; Xu et al., 2006; Ajithkumar and Panneerselvam, 2014). To alleviate the damage induced by ROS, plants are capable of balancing electron transfer between light absorption and light utilization via energy dissipation mechanisms. Moreover, the majority of plants are equipped with corresponding antioxidant mechanisms, encompassing both enzymatic and non-enzymatic antioxidant systems. The enzymatic antioxidant system predominantly encompasses superoxide dismutase (SOD), peroxidase (POD), and catalase (CAT), whereas non-enzymatic antioxidants mainly consist of anthocyanins, ascorbic acid, and tocopherol (Moharramnejad et al., 2019). In addition to the antioxidant system, the osmotic adjustment system–such as soluble sugars, soluble proteins, and proline–also serves as a crucial mechanism by which plants resist and adapt to drought stress (Malviya, 2015).

The advancement of high-throughput sequencing technology has significantly enhanced our capacity to investigate plant drought resistance mechanisms at the molecular level. Among these approaches, transcriptome sequencing (RNA-Seq) is the most widely used and direct method. It enables a comprehensive characterization of gene expression in plants under drought stress by identifying up-regulated and down-regulated genes. For instance, transcriptome analysis of maize subjected to drought stress has revealed that differentially expressed genes (DEGs) that are upregulated and downregulated are enriched in specific biological processes, including carbohydrate (CHO) metabolism, signal transduction, abscisic acid (ABA) signaling, brassinosteroid biosynthesis, and stomatal movement. This finding indicates that drought resistance is closely associated with differential gene expression (Jiang et al., 2023). Previous transcriptomic analyses of maize, tomato, and sugarcane under drought stress have shown that transcription factors, along with genes associated with photosynthesis–antenna proteins, nitrogen metabolism, the MAPK signaling pathway, purine metabolism, starch and sucrose metabolism, and β-alanine metabolism, are all involved in the response to drought conditions (Jiang et al., 2023; Li et al., 2023; Shu et al., 2024). Transcriptome analysis of peony under drought stress demonstrated that a multitude of genes contribute to enhancing drought tolerance through the regulation of ROS systems, chlorophyll degradation, photosynthetic capacity, fatty acid metabolism, and proline metabolism (Zhao et al., 2019). So far, RNA-Seq has facilitated the discovery of numerous key gene families associated with drought resistance. Transcription factors such as DREB (Venkat and Muneer, 2025), NAC (Saidi et al., 2025), and MYB (Saidi et al., 2025) have been identified as master regulators in modulating the gene regulatory network associated with drought resistance, whereas functional genes encoding LEA proteins (*PmLEA25*) (Wang et al., 2024), aquaporins (*TIP1-1*) (Yüksel Özmen et al., 2025), antioxidant enzymes (Liu et al., 2025), and proline synthetases are directly involved in executing key physiological mechanisms underlying drought tolerance.

Cranberry (*Vaccinium macrocarpon*), an evergreen, low-growing shrub belonging to the *Ericaceae* family, bears red spherical berries. Owing to its abundant content of vitamins, minerals, flavonols, proanthocyanidins, and resveratrol, it is frequently regarded as a “superfruit” (Teleszko, 2011). Amid the escalating demand for health foods among consumers, cranberries, recognized as a fruit abundant in nutrients and conducive to health, exhibit substantial market potential. In recent years, the cranberry industry in China has witnessed remarkable growth, evolving into an emerging specialized fruit industry. As of 2023, the cranberry cultivation area in the country has reached 4,200 acres, yielding 2,700 tons, establishing it as the largest cranberry cultivation base in Asia. Cranberries are indigenous to North America and predominantly thrive in moist evergreen habitats, including marshlands, mountain meadows, and forests. These habitats necessitate stringent water conditions, as both excessive aridity and waterlogging can be detrimental to cranberry growth. At present, research on cranberries mainly centers around the fruit composition and the associated healthcare and medicinal properties. Significantly less emphasis, however, has been placed on the physiological aspects of cranberry cultivation. Numerous studies have shown that, owing to the shallow root system of cranberries, an excessive amount of moisture frequently gives rise to root rot and a decline in fruit quality. On the contrary, water scarcity leads to smaller berries, inadequate plant coverage, and even plant death. Moreover, cranberry plants commonly encounter drought stress during the summer months (Erndwein et al., 2023). Previous studies have investigated the physiological responses of two major cranberry cultivars, ‘Ben Lear’ and ‘Stevens’, to drought stress under greenhouse conditions. The findings indicated that the transpiration rate and stomatal conductance of cranberries started to decline on the 9th day of drought exposure, while the photosynthetic rate decreased significantly on the 13th day of drought. By the 15th day of drought, both the leaf water content and the performance of the photosystem decreased substantially. It was noted that cranberries are most susceptible to drought stress during the vegetative growth stage, and stomatal conductance is the most sensitive parameter (Cesped-Ruiz and Huang, 2004). Nevertheless, the remaining physiological responses of cranberries under drought stress have not been comprehensively analyzed. Moreover, there are no published reports on the molecular mechanism underlying cranberry drought resistance. In this study, to elucidate the physiological and molecular responses of cranberry to drought stress, we systematically measured photosynthetic oxygen evolution, chlorophyll fluorescence parameters, osmoregulatory substances, and antioxidant enzyme activities; observed stomatal morphological characteristics and leaf anatomical structure; and performed transcriptome sequencing analysis sequencing analysis under varying levels of drought treatment. This research is expected to enhance the theoretical comprehension of plant drought resistance mechanisms and offer a theoretical foundation for the cultivation and utilization of cranberries.

## 2 Materials and methods

### 2.1 Plant materials and experimental setup

The experimental materials were three-year-old cranberry ‘Bain 11’ plants cultivated in pots. The cultivation substrate consists of a 2:1:1:4 mixture of peat, perlite, coconut coir, and garden soil. The soil acidity was adjusted to pH 5.5 by using sulfur powder. The experiment was conducted in a solar greenhouse at Jilin Agricultural University between June and July 2022. The greenhouse utilized natural light and ambient temperature conditions and was equipped with rain-sheltering facilities. During the experimental period, the light intensity in the greenhouse was maintained at 750 ± 180 μmol·m^−2^·s^−1^, with a photoperiod of 15 hours of light and 9 hours of darkness. The relative air humidity was 65% ± 10%, and the average daily temperature was 26 ± 4 °C. During the fruit expansion phase, a potted water control experiment was executed. This experiment incorporated four treatments. The control group (CK) sustained the soil water content at 85% of the field capacity. For the experimental groups, three levels of drought stress were imposed. D1 denoted mild drought stress (water withholding for 3 days, with the soil water content maintained at 65%–75% of the field capacity), D2 signified moderate drought stress (water withholding for 6 days, with the soil water content maintained at 45%–60% of the field capacity), and D3 represented severe drought stress (water withholding for 9 days, with the soil water content maintained at 25%–40% of the field capacity). Each treatment group was established with 20 potted plants (one plant per pot), representing 20 biological replicates. For the determination of physiological, biochemical, and anatomical parameters, three biological replicates were randomly selected from each group, and each parameter was measured three times to obtain the mean value for statistical analysis.

### 2.2 Physiological and biochemical parameters

Leaf water content was determined following the method described by Zhao et al (Zhao et al., 2019). Chlorophyll a, b, and total chlorophyll were extracted from leaves using 80% acetone and quantified by spectrophotometry, with concentrations calculated as outlined in reference (Arnon, 1949). Hydrogen peroxide (H_2_O_2_), malondialdehyde (MDA), and CAT levels were assayed using commercial kits (Solarbio, China) in accordance with the manufacturer’s protocols. SOD activity was measured by the nitroblue tetrazolium (NBT) photoreduction method (Khan et al., 2022). When determining the above indicators, three biological replicates were randomly selected from each group. For each biological replicate, each indicator was measured three times technically, and the average value was taken for statistical analysis.

### 2.3 Measurement of photosynthetic parameters

The amount of photosynthetically released oxygen was determined using an Oxytherm+P liquid-phase oxygen electrode system (Hansatech, UK) on a sunny morning, with a photosynthetic photon flux density of 1200 μmol·m^-2^·s^-1^ and temperature maintained at ambient conditions. Healthy, mature leaves were suspended in the reaction chamber with their upper surfaces perpendicular to the light source. The rate of photosynthetic oxygen release per unit leaf fresh weight was derived from the linear phase of the oxygen evolution curve following the onset of stable illumination.

The rapid fluorescence induction kinetics (OJIP transient) were recorded with a Handy-PEA fluorometer (Hansatech, UK). Plants were dark-adapted for 30 minutes prior to the measurement of chlorophyll fluorescence parameters. A pulse of light with an intensity of 3000 μmol·m^-2^·s^-1^ was applied to induce fluorescence, and the measurement duration was set to 1 s. The obtained OJIP fluorescence induction kinetics curves were analyzed using the JIP-test method described by Strasser et al (Strasser et al., 2000, 2004).

The FMS-2 pulse-modulated fluorometer (Hansatech, UK) was used to determinate the chlorophyll fluorescence quenching parameters. The measurement protocol was as follows: Following full dark adaptation of the leaves, they were securely clamped into the leaf clip. First, the measuring light was activated to determine the minimum fluorescence yield (Fo) of the dark-adapted state. Subsequently, a saturating pulse of light was applied to obtain the maximum fluorescence yield (Fm). After the fluorescence signal stabilized following a decline, actinic light was applied to initiate photosynthetic activity. As photosynthesis proceeded, the fluorescence level decreased and eventually reached a steady state; the fluorescence value at this point was recorded as Fs. A second saturating pulse was then applied under continuous actinic illumination to measure the maximum fluorescence yield (Fm’) during light adaptation—note that the actinic light remained on during this step. Upon completion of Fm’ measurement, the actinic light was turned off and far-red illumination was initiated to activate photosystem I (PSI), thereby oxidizing the electron acceptors of photosystem II (PSII). Under these conditions, the minimum fluorescence yield (Fo’) was measured, representing the state where all PSII reaction centers are fully open during light adaptation. Chlorophyll fluorescence quenching parameters were subsequently calculated using the recorded values according to the following equations (Borsani et al., 2001):


Maximum quantum yield of PSII under light adaptation: Fv'/Fm' = (Fm'−Fo')/Fm'

Proportion of open PSII reaction centers under light adaptation: qP = (Fm'−Fs)/(Fm'−Fo')

Actual photochemical efficiency of PSII under light adaptation:ΦPSII = (Fm'−Fs)/Fm'

Non−photochemical quenching under light adaptation: NPQ = (Fm−Fm')/Fm'

Fluorescence decline ratio under light adaptation: Rfd = (Fm−Fs)/Fs

When measuring the above indicators, three biological replicate samples were randomly selected from each group. For each biological replicate, each indicator was measured three times technically, and the average value was used for subsequent statistical analysis.

### 2.4 Observation of stomatal morphological characterization

For stomatal morphological characterization, fully expanded leaves were sampled between 8:00 and 9:00 AM. Impressions of the abaxial leaf surface were obtained by applying clear nail polish, allowing it to dry, and subsequently lifting the resulting film to prepare microscopic specimens (Stirbet et al., 2018). Stomatal characteristics, including size, area, density, and the proportion of open stomata (calculated as the number of open stomata divided by the total number of stomata), were examined using Nikon DS-Ri2 Microscope (Nikon, Japan) and quantified using NIS-Elements F software, with average values derived from ten fields of view per parameter. For each group, three biological replicates were randomly selected (one plant per pot). One section was prepared for each biological replicate, and 12 independent fields of view were randomly selected from each section for measurement. The results are expressed as the mean ± standard deviation.

### 2.5 Observation of leaf microstructure

The anatomical features of the leaves were examined using a paraffin sectioning technique. Mature leaves (the second to third pairs from the apex of sun-exposed branches) were randomly sampled and sectioned into 1 cm × 0.5 cm segments along the midrib. Following fixation in FAA, 10 μm thick sections were prepared, stained with safranin and fast green, and mounted with neutral balsam. Leaf tissue layers—including the upper and lower epidermis, palisade mesophyll, and spongy mesophyll—were imaged using a DS-Ri2 microscope (Nikon, Japan), and their thicknesses were quantified with NIS-Elements F software. Measurements represent mean values from 12 independent fields of view. For each group, three biological replicates were randomly selected (one plant per pot). One section was prepared for each biological replicate, and 12 independent fields of view were randomly selected from each section for measurement. The results are expressed as the mean ± standard deviation.

The ultrastructural analysis of leaf tissues was performed using a modified version of the method described by Khan et al (Khan et al., 2022). Cut 1 mm × 3 mm strips from cranberry leaves, avoiding the main veins and immediately immerse them in a pre-cooled glass vial containing 3.5% glutaraldehyde. Apply vacuum infiltration until the tissue strips fully submerge, then fix at 4 °C for 24 h. Rinse the samples 4–5 times with 0.1 mol/L phosphate buffer (pH 7.2), followed by post-fixation with 1% osmium tetroxide at 4 °C for 4 h. Repeat rinsing 4–5 times with the same buffer. Dehydrate through an ascending ethanol series (30% to 100%), infiltrate with Spurr’s resin, and embed the samples. Polymerize at 60 °C for 24 h. Section the embedded tissues into ultrathin slices (500–700 Å) using an LKB ultramicrotome. Collect the sections on 250-mesh copper grids and stain sequentially with uranyl acetate and lead citrate, each for 30 min. Examine under a JEM-1200EX transmission electron microscope (NEC Corporation, Japan) operated at 80 kV. Three biological replicates (one plant per pot) were randomly selected from each group, and three sections were observed for each biological replicate.

All the above-mentioned measurement indicators were tested by one-way analysis of variance. A difference was considered statistically significant when p < 0.05.

### 2.6 RNA-seq and data analysis

Leaf samples were collected from cranberry plants (CK, D1, D2, D3) subjected to different treatments, rapidly frozen in liquid nitrogen, and stored for subsequent analysis. Three biological replicates were established for each experimental group. Total RNA was extracted using the CTAB method. RNA purity and concentration were assessed using a microvolume ultraviolet spectrophotometer, with acceptable purity criteria defined as OD260/OD280 ratios between 1.8 and 2.0 and OD260/OD230 ratios greater than 2.0. RNA integrity was further verified by 1.2% agarose gel electrophoresis to ensure sample quality met the requirements for subsequent experiments. Qualified RNA samples were subjected to transcriptome sequencing, which was performed by OE Biotech Co., Ltd. After filtering out low-quality reads from the raw sequencing data, *de novo* transcriptome assembly was carried out using Trinity software. A non-redundant set of unigene sequences was generated through CD-HIT clustering to eliminate sequence redundancy. A cDNA library was constructed based on the unigene sequences and subjected to quality evaluation. Differentially expressed genes (DEGs) were functionally annotated and classified using the NR, KOG, GO, and KEGG databases. Finally, KEGG pathway enrichment analysis was performed on the target DEGs. Differentially expressed genes were identified with |log_2_FC| > 1 and FDR < 0.05.

### 2.7 Quantitative real-time polymerase chain reaction verification

Total RNA was reverse-transcribed into cDNA using the PrimeScript RT kit with gDNA Eraser (TaKaRa, Japan). To validate the sequencing accuracy, the expression levels of six drought-responsive genes were assessed via quantitative reverse transcription PCR (qRT-PCR) using TB Green^®^ Premix Ex Taq™ (Tli RNaseH Plus) on the ABI StepOnePlus™ Real-Time PCR System (ABI, State of California, USA). The PCR program was as follows: an initial denaturation at 94 °C for 30 s, followed by denaturation at 94 °C for 5 s, and then 40 cycles consisting of annealing at 60 °C for 30 s and extension at 95 °C for 15 s. The SAND gene from cranberry served as the reference, and all primers were designed with Primer Express 3.0 software and synthesized by Suzhou Genewiz Biotechnology Co., Ltd. The specific genes and corresponding primer sequences are provided in **Supplementary Table 1**. Each reaction was repeated three times. The relative expression levels of genes were calculated using the 2^-ΔΔCt^ method.

### 2.8 Data processing

The measured data were statistically analyzed using SPSS 25.0. One-way analysis of variance (ANOVA) with the least significant difference (LSD) method was employed, and the significance level was set at P < 0.05. Subsequently, charts and graphs were generated using Excel 2024.

## 3 Results

### 3.1 The influence of drought stress on the photosynthetic performance of cranberries

**Figure 1:**
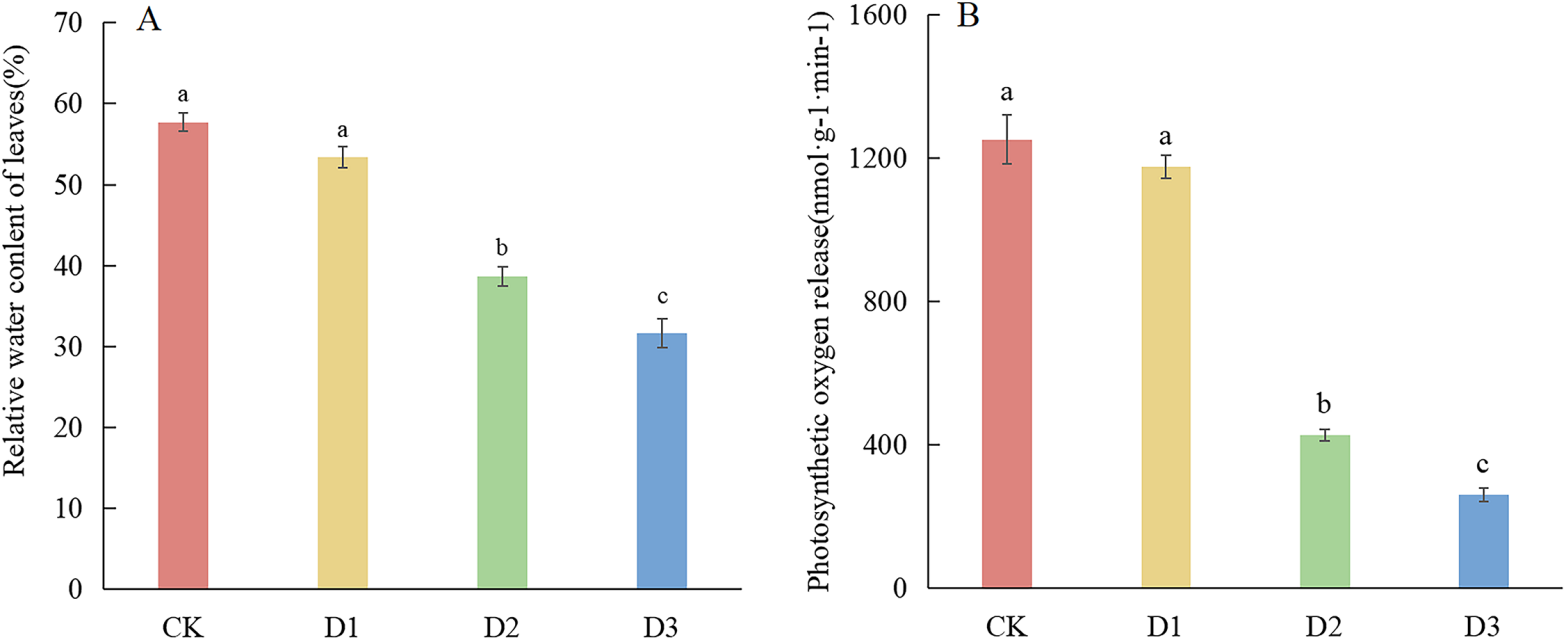
Effects of different degrees of drought stress on leaf relative water content and photosynthetic oxygen evolution of cranberry leaves. **(A)** Leaf relative water content; **(B)** Photosynthetic oxygen evolution rate.

The findings of this study suggest that increasing drought stress leads to a progressive decline in water content in cranberry leaves. Compared with CK, the water content in the cranberry leaves under D2 and D3 treatments decreased significantly by 33% and 45%, respectively (**Figure 1A**). The amount of photosynthetically released oxygen in leaves and the leaf water content exhibited a similar trend: both were markedly reduced under D2 and D3 conditions, declining by 69% and 79%, respectively (**Figure 1B**). Chlorophyll serves as an indispensable pigment for photosynthesis in plants. Measurements of chlorophyll content showed no significant difference under D1 and D2 treatments relative to CK;. however, a substantial 43% reduction was observed (**Table 1**).

**Table 1:** Effects of drought stress on pigment content in leaves of cranberry.

Treatment	Chlolophyll a content (mg/g)	Chlolophyll b content (mg/g)	Total chlolophyll content (mg/g)
CK	0.98 ± 0.06 a	0.41 ± 0.02 b	1.38 ± 0.07 a
D1	0.97 ± 0.03 a	0.43 ± 0.02 ab	1.41 ± 0.03 a
D2	1.01 ± 0.04 a	0.47 ± 0.05 a	1.48 ± 0.05 a
D3	0.52 ± 0.03 b	0.27 ± 0.03 c	0.79 ± 0.03 b

**Figure 2:**
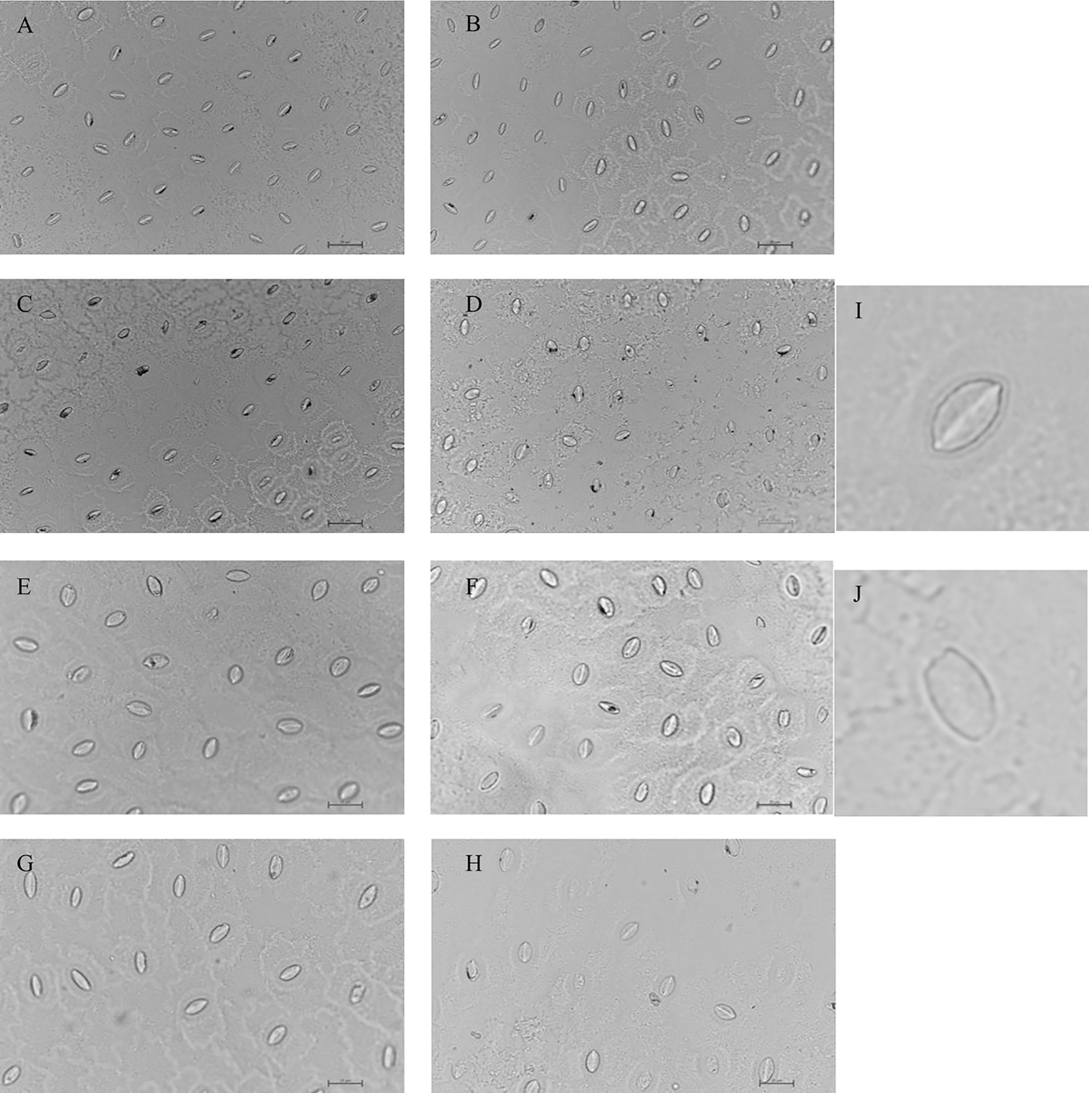
Effects of drought stress on stomatal characteristics of cranberry leaves(surveyor’s rod is 25μm). Regions **(A–D)** represent a 40-fold magnification of the visual field, while regions **(E–H)** represent a 60-fold magnification, with both sets corresponding sequentially to CK, D1, D2, and D3. Region **(I)** shows open stomata, whereas Region **(J)** shows closed stomata.

Stomata, serving as the primary pathway for carbon dioxide entry into leaves, are closely associated with photosynthesis through their morphological characteristics, such as size, density, and opening ratio. To investigate the impact of drought stress on these traits, stomatal characteristics in cranberry leaves were observed and measured under varying levels of water deficit. Compared with CK, drought did not significantly influence the stomatal length, width, or area of cranberry leaves, but stomatal density and stomatal opening ratio exhibited a significant decrease under D2 and D3 treatments, which was consistent with the decline in photosynthetically released oxygen under the same treatments (**Figure 2**; **Table 2**).

**Table 2:** Effects of drought stress on stomatal characteristics of cranberry leaves.

Treatment	Stomata length (μm)	Stomata width (μm)	Stomata area (μm^2^)	Stomata density (number/mm^2^)	Stomatal opening ratio (%)
CK	11.99 ± 0.29 a	6.64 ± 0.28 a	61.98 ± 4.62 a	866.97 ± 15.09 a	76.91 ± 5.54 a
D1	11.21 ± 0.46 ab	5.66 ± 0.11 b	55.01 ± 2.67 a	838.45 ± 9.88 a	74.41 ± 7.34 a
D2	10.78 ± 0.15 b	5.37 ± 0.26 b	45.64 ± 1.75 b	701.56 ± 26.14 bc	37.41 ± 5.13 b
D3	11.31 ± 0.38 a	6.36 ± 0.14 a	57.16 ± 1.32 a	647.49 ± 15.09 c	26.11 ± 4.84 c

**Figure 3:**
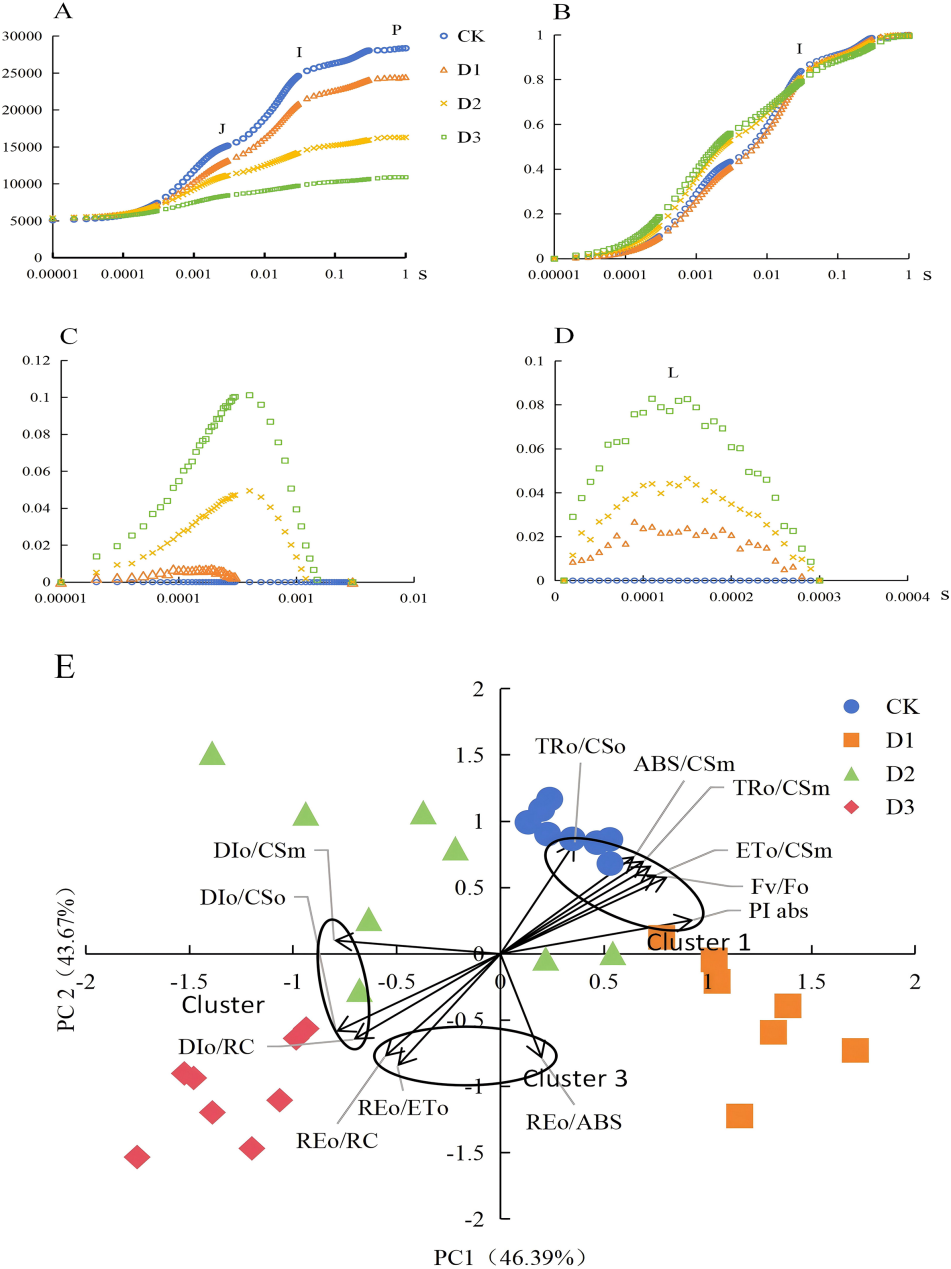
Effects of drought stress on chlorophyll fluorescence characteristics and PCA clustering analysis of cranberry. **(A–D)** Chlorophyll fluorescence kinetic curves (O-J-I-P curve, standardized O-J-I-P curve, O-J standardized curve, O-K standardized curve); **(E)** PCA clustering analysis of chlorophyll fluorescence parameters.

Chlorophyll fluorescence technology enables a comprehensive analysis of the photosynthetic electron transfer status in leaves and finds extensive applications in diverse experiments related to biotic or abiotic stress. In this study. the results revealed that varying degrees of drought stress treatment exerted a significant influence on the OJIP curve of chlorophyll fluorescence in cranberries. Specifically, as the intensity of drought stress increased, the fluorescence value at point P significantly declined (**Figure 3A**). To facilitate a clear comparison of fluorescence kinetics curves across different treatments and varieties, the rapid chlorophyll fluorescence induction curves were normalized using (Fm - Fo) to obtain the relative variable fluorescence, Vt. As shown in **Figure 3B**, the J point of the OJIP curve in the D2 and D3 treatments was significantly elevated. Moreover, these treatments exhibited distinct positive peaks in chlorophyll fluorescence yield at 300 μs (K point) and 150 μs (L point) (**Figures 3C, D**). While under condition D1, the chlorophyll fluorescence yield reached a peak at the L point, with minimal influence on the K point (**Figures 3C, D**). The JIP-test method can efficiently analyze crucial information regarding the photosynthetic electron transport chain in plants under drought stress conditions. Following the statistical analysis of the selected JIP-test parameters using principal component analysis (PCA), it was revealed that multiple parameters formed three distinct clusters (**Figure 3E**). The parameters within Cluster 1 characterize the photosynthetic performance index of PSII, as well as the light energy capture and electron transfer stages; those in Cluster 2 depict the energy dissipation stage; and those in Cluster 3 represent the performance at the end of the electron transfer chain. PC1 is associated with PSII activity, where higher values signify enhanced PSII performance; PC2 is related to PSI activity, with higher values indicating greater PSI activity. Cluster analysis revealed that CK and D1 were predominantly distributed around Cluster 1, whereas D2 was distributed between Clusters 1 and 2, and D3 was mainly distributed between Clusters 2 and 3. By integrating the significance of these parameters, we observed that the PSII performance under condition D1 was slightly higher than that of CK, whereas under condition D2, the PSII performance was lower than that of CK, accompanied by a marked increase in dissipation capacity. Under condition D3, the performances of both PSII and PSI were the poorest. To further validate the above conclusions, the FMS-2 portable pulse-modulated fluorometer was used to investigate cranberry leaves under drought stress, benefiting from its ability to effectively distinguish fluorescence signals from ambient light interference. As presented in **Table 3**, compared with CK, the reduction in Fv’/Fm’ under D1 was minimal, whereas significant decreases of 37% and 54% were note D2 and D3, respectively. The trends of ΦPSII and Rfd under different stress conditions were consistent with those of Fv’/Fm’. In contrast, the qP decreased significantly by 47% and 51% under conditions D2 and D3, respectively, while the NPQ only significantly increased under condition D2. Additionally, the decline in chlorophyll content under D3 was consistent with the severely impaired PSII and PSI performance under this treatment.

**Table 3:** Effects of different degrees of drought stress on fluorescence quenching of cranberry PS II.

Treatment	Fv’/Fm’	qP	ФPSII	NPQ	Rfd
CK	0.81 ± 0.02 a	0.93 ± 0.02 a	0.75 ± 0.02 a	0.65 ± 0.11 b	5.51 ± 0.07 a
D1	0.79 ± 0.02 a	0.95 ± 0.01 a	0.75 ± 0.01 a	0.76 ± 0.05 b	5.66 ± 0.35 a
D2	0.51 ± 0.03 b	0.49 ± 0.05 b	0.23 ± 0.03 b	1.53 ± 0.02 a	2.36 ± 0.11 b
D3	0.37 ± 0.02 c	0.46 ± 0.02 b	0.17 ± 0.02 c	0.66 ± 0.13 b	1.01 ± 0.19 c

### 3.2 The response of the cranberry leaf anatomical structure to drought stress

**Figure 4:**
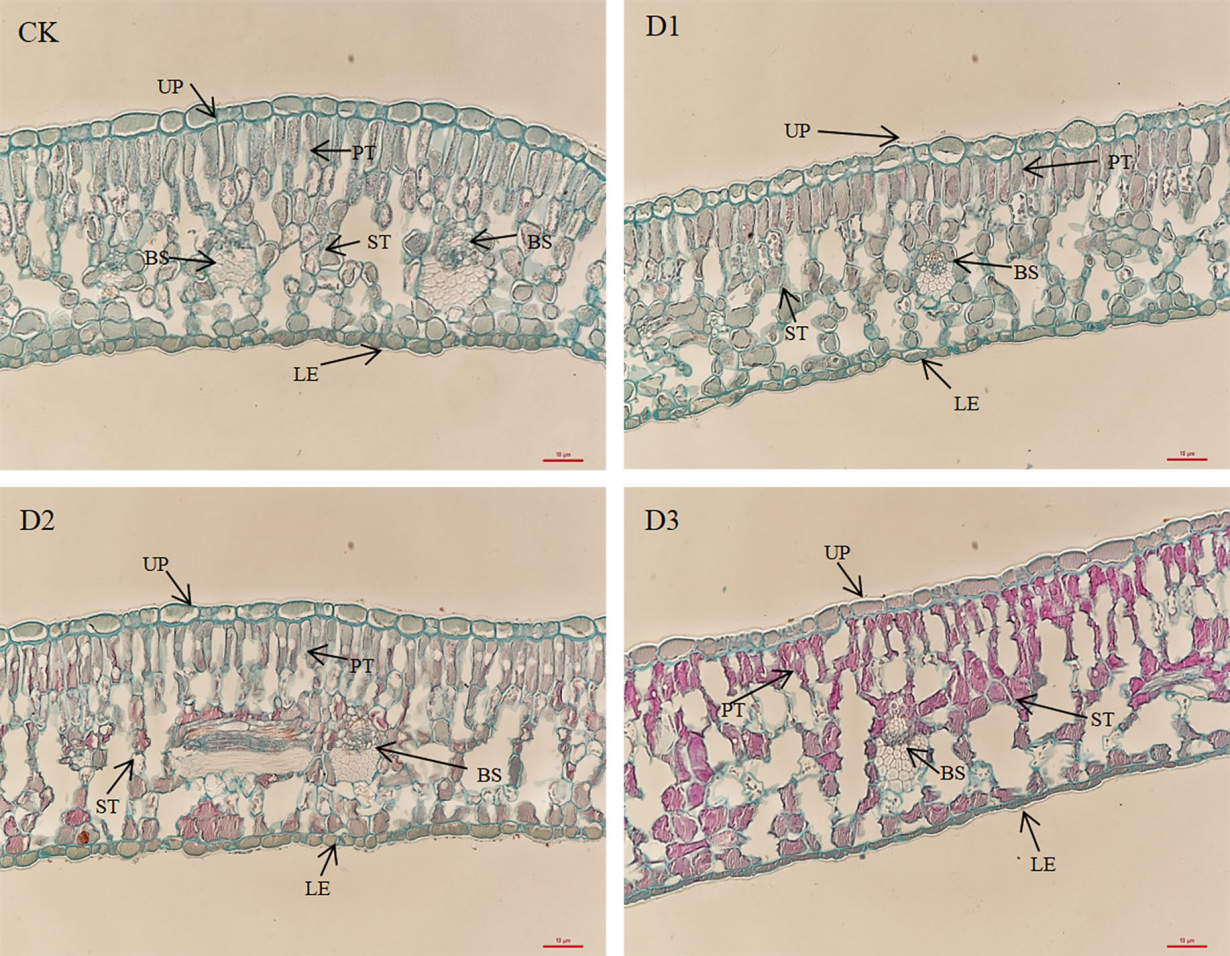
Effects of drought stress on leaf anatomy of cranberry(surveyor’s rod is 10 μm). UP, upper epidermis; PT, palisade tissue; ST, spongy tissue; LE, lower epidermis; BS, bundle sheath.

This study selected upper epidermal cells, lower epidermal cells, palisade tissue cells, and spongy tissue cells from the leaf anatomical structure as observation indicators to conduct a comparative analysis of changes in the anatomical structure of cranberry leaves under varying degrees of drought stress. As illustrated in **Figure 4** and **Table 4**, in the CK treatment, the upper and lower epidermal cells of cranberry leaves are unilayered, elongated, and oval in shape, arranged in a tightly packed manner. The palisade tissue consists of two layers of columnar cells that are closely aligned. The spongy mesophyll is located beneath the palisade layer and adjacent to the lower epidermis, exhibiting irregular shapes with a loose arrangement pattern. Compared with CK, no significant morphological changes were detected in the various cellular structures of cranberry leaves under the D1 treatment. Under the D2 treatment, the thickness of palisade tissue layer and spongy tissue layer exhibited a reduction of approximately 20.3% and 10.5%, respectively, while the intercellular space began to increase. However, in the D3 treatment, specific palisade cells display cracking and disorganization, while the intercellular spaces of spongy mesophyll tissue become further enlarged. Moreover, the thickness of the upper epidermal cells and lower epidermal cells also shows a significant reduction, by 41.8% and 38.9%, respectively. This progressive damage to leaf anatomical structure was consistent with the progressive decline in leaf water content and photosynthetic performance with increasing drought stress.

**Table 4:** Effects of drought stress on leaf anatomy of cranberry.

Treatment	Upper epidermis (μm)	Palisade tissue (μm)	Spongy tissue (μm)	Lower epidermis (μm)
CK	9.38 ± 0.43 a	34.94 ± 1.15 a	52.28 ± 1.72 a	6.73 ± 0.05 a
D1	8.94 ± 0.54 a	37.01 ± 0.19 a	48.04 ± 2.01 b	5.27 ± 0.14 a
D2	7.19 ± 0.67 b	30.23 ± 1.41 b	46.78 ± 1.49 bc	5.22 ± 0.05 a
D3	5.46 ± 0.33 c	29.53 ± 0.74 b	43.28 ± 1.29 c	4.11 ± 0.11 b

**Figure 5:**
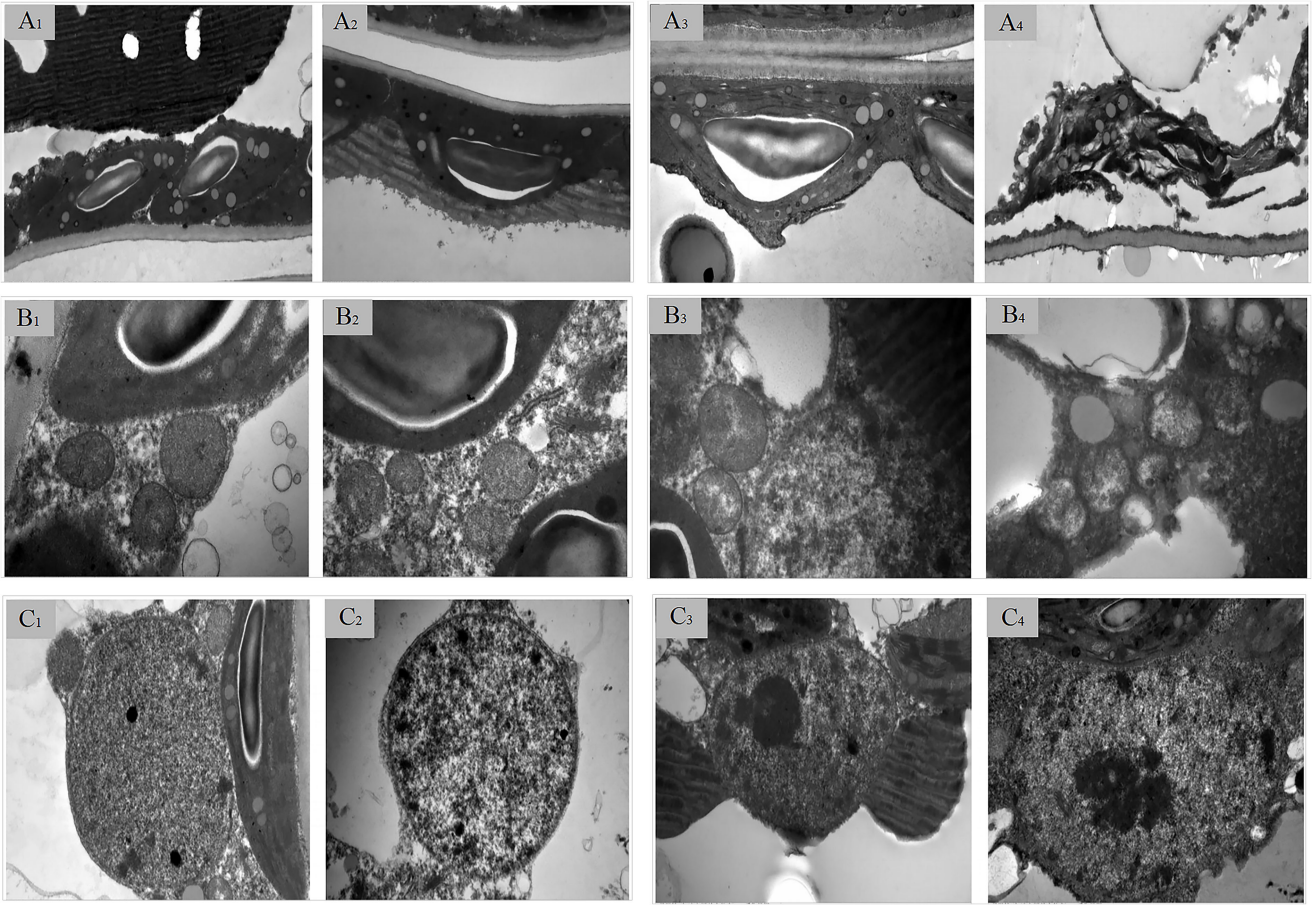
Effects of drought stress on the ultra microstructure of cranberry leaves. **(A–C)** represent the ultrastructure of chloroplasts, mitochondria, and the cell nucleus respectively. The subscripts 1–4 correspond to CK, D1, D2, and D3 respectively. The scale of **(A, C)** is 2 μm, while the scale of **(B)** is 500 nm.

The impact of drought on plant leaf structure is multifaceted and occurs at multiple levels, inducing significant adaptive or detrimental alterations from the epidermis and mesophyll to subcellular ultrastructures. In CK, chloroplasts exhibit an elongated oval shape, closely adhering to the cell membrane in a single layer, possessing distinct and intact membrane structures, compact thylakoid lamellae, with osmiophilic granules presenting low abundance, small individual size, and scattered distribution throughout the stroma without obvious aggregation. Mitochondria appear circular, with intact double membranes and dense, compact contents. Nuclei are spherical, displaying complete structures, clear double membranes, and a homogeneous distribution of nuclear substances (**Figure 5**-A_1_, B_1_, C_1_). Under D1 condition, the number of osmiophilic granules within chloroplasts increases and becomes concentrated, showing a noticeable enrichment trend with obvious local accumulation compared with the CK group, while the morphology remains unchanged. The membrane structure of mitochondria does not exhibit obvious alterations; however, the contents become sparse. The structure of the nucleus remains intact, yet the nuclear substances are diminished (**Figure 5**-A_2_, B_2_, C_2_). Under D2 condition, chloroplasts expand, the outer membrane becomes indistinct and separates from the cell membrane, osmiophilic granules enlarge, accompanied by increased particle volume, partial fusion of adjacent granules, and further intensified aggregation distribution, and the thylakoid lamellae are loosely arranged with noticeable gaps. The contents of mitochondria significantly decrease, leading to the formation of cavities, and the outer membrane becomes blurred. The nucleus undergoes significant shrinkage, adopts an irregular shape, and the nuclear membrane deforms, resulting in a blurred outer membrane (**Figure 5**-A_3_, B_3_, C_3_). Under D3 condition, chloroplasts completely detach from the cell membrane, resulting in structural breakdown of the membrane and the formation of numerous internal cavities; the thylakoid lamellae become unrecognizable. Additionally, the outline of mitochondria becomes indistinct, the membrane structure ruptures, and cellular contents leak out, creating multiple cavities. The nucleus also loses its normal architecture, with the nuclear membrane breaking and nuclear substances spilling out, indicating a trend toward disintegration (**Figure 5**-A_4_, B_4_, C_4_). These alterations suggest that as drought stress intensifies, the structures of chloroplasts, mitochondria, and nuclei in cranberry leaves undergo progressive damage, leading to significant impairment of their metabolic functions. The progressive destruction of the ultrastructure of chloroplasts is highly consistent with the gradual decline of the above-mentioned PSII performance parameters (Fv’/Fm’, ΦPSII, qP) and the significant increase of the J and K points in the OJIP curve along the stress gradient.

### 3.3 The influence of drought stress on antioxidant enzyme activities

**Figure 6:**
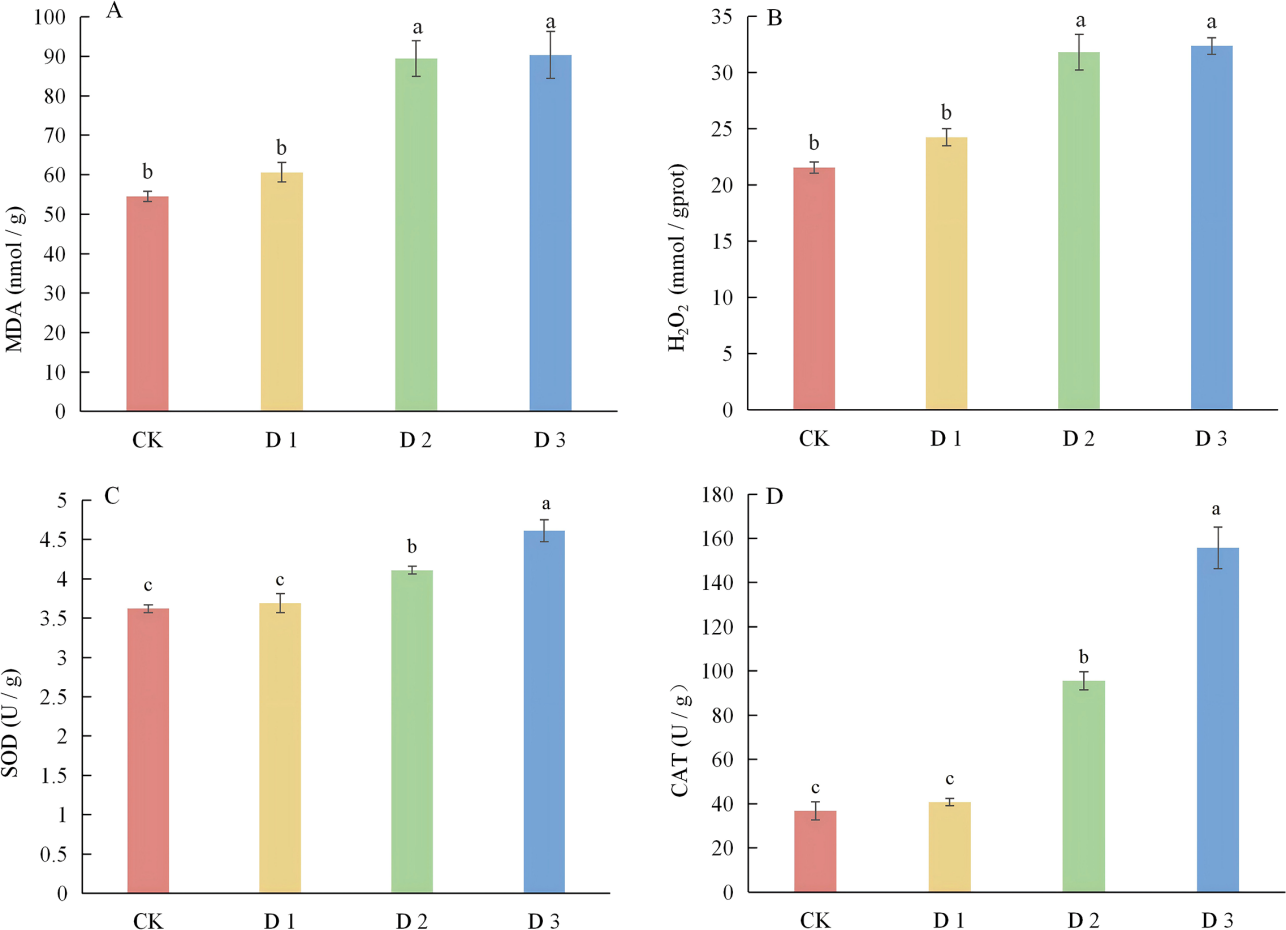
Effects of different drought stress on cranberry MDA, H_2_O_2_, SOD and CAT. **(A)** MDA; **(B)** H_2_O_2_; **(C)** SOD; **(D)** CAT.

MDA (malondialdehyde) serves as a key product of lipid peroxidation occurring within cell membranes. Lipid peroxidation proceeds in a progressive manner, leading to the degradation of cell membranes and ultimately culminating in cell death. As depicted in **Figure 6A**, with the progressive intensification of drought stress, the MDA content in cranberry leaves exhibits a gradual upward trend. In comparison with CK, the MDA content in cranberry leaves under D1 treatment increased by merely 11% and did not exhibit a significant difference. In D2 treatment, the MDA content in cranberry leaves increased significantly by 64%. Similarly, in D3 treatment, the MDA content increased significantly by 66%. However, no significant difference was observed between the D2 and D3 treatments. H_2_O_2_ serves as a crucial representative of reactive oxygen species. Both biotic and abiotic stresses are capable of triggering the production and accumulation of H_2_O_2_ within plant cells. Under drought stress conditions, the variation trend of H_2_O_2_ in cranberry leaves is analogous to that of MDA content. In comparison CK, the H_2_O_2_ content in cranberry leaves under D1 treatment exhibited a 12.5% increase, yet this increase was not statistically significant. Under D2 treatment, the H_2_O_2_ content significantly rose by 47.6%. Moreover, under D3 treatment, it significantly increased by 50.2%. Notably, there was no significant difference in the H_2_O_2_ content between the D2 and D3 treatments (**Figure 6B**).

When plants are subjected to drought stress, owing to the accumulation of reactive oxygen species within the plant, the leaves safeguard themselves by modulating the content and activity of a series of antioxidant enzymes. In this research, the impacts of varying degrees of drought stress on the activities of cranberry antioxidant enzymes SOD and CAT were quantified. The trends of the activities of these two antioxidant enzymes were relatively consistent. Specifically, the activity of the antioxidant enzymes was directly proportional to the degree of drought stress (**Figures 6C, D**). The activities of SOD and CAT in cranberry leaves under D1 treatment were essentially consistent with those of CK. When compared to CK, the activities of SOD and CAT increased by 13.5% and 160% respectively under D2 treatment. In D3 treatment, the activities of SOD and CAT significantly increased by 27.3% and 324% respectively. Evidently, CAT might play a more crucial role than SOD when cranberries respond to reactive oxygen species induced by drought stress. At the same time, we found that the increase in H_2_O_2_ content was consistent with the changing trends of SOD and CAT activities, and the accumulation of MDA was in line with the changes in H_2_O_2_ content, indicating that the balance between the generation and clearance of reactive oxygen species was directly related to the degree of cell membrane damage.

### 3.4 Sequence analysis, transcript assembly, and gene functional annotation

To investigate the molecular response of cranberry to drought stress, high-throughput RNA-seq was conducted on leaf samples collected from four experimental groups subjected to drought conditions. Following the removal of low-quality reads from the raw sequencing data, the Q30 value (percentage of bases with a Phred score ≥ 30) exceeded 95% across all treatment groups, and GC content remained stable at approximately 46% (**Supplementary Table 2**), indicating high data quality suitable for downstream analyses. Transcript assemblies were generated using the Trinity software with paired-end reads. Subsequent clustering and redundancy reduction via CD-HIT yielded a final set of 58,020 unigenes, which served as the reference sequences for functional annotation.

**Figure 7:**
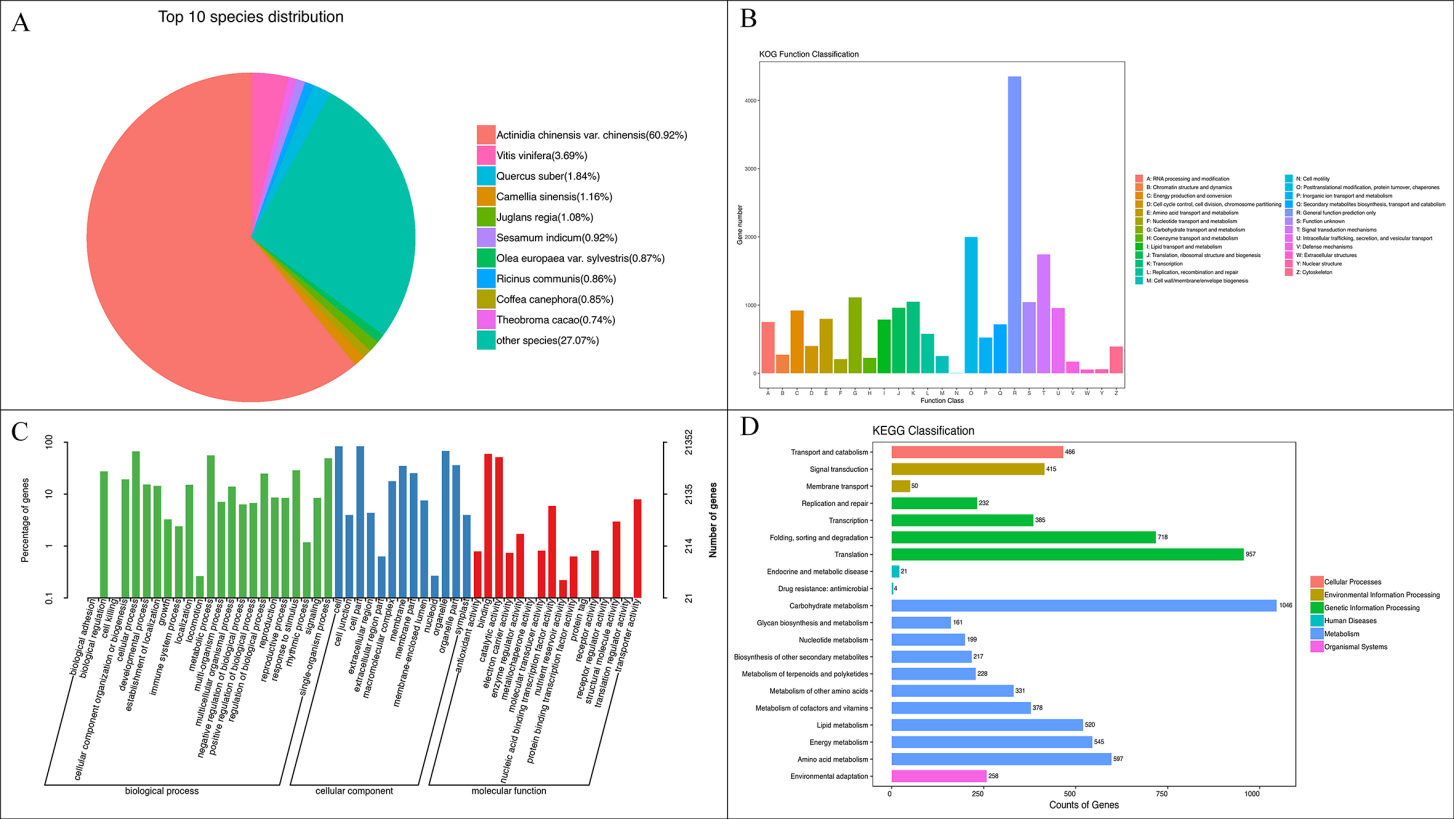
Functional annotation and classification of genes from transcriptomic data. **(A)** NR Top10 Species Distribution.Fan shapes of different colors represent the proportion of species annotated. **(B)** KOG Classification of gene functions in the database. X-axis represents the functional classification, Y-axis represents the number of genes. **(C)** Gene Function Annotation of GO Database. Horizontal axis representation GO functional classification, left vertical axis indicates the number gene annotations, right vertical axis represents the number gene annotations to this class. **(D)** KEGG pathway annotation information at Level2 level. X-axis represents the number of genes,Y-axis represents the names of Level 2 pathways.

The obtained unigenes were compared with four major databases (NR, KOG, GO, and KEGG) to obtain annotation information in each database. There were 30,910 entries in the NR database, 21,352 entries in the GO database, 18,183 entries in the KOG database, and 6,133 entries in the KEGG database (**Supplementary Table 3**).The homology analysis of species in the NR database shows that the most homologous genes are found in Chinese kiwi (18,821, 60.92%), followed by grape (1,139, 3.69%), and other species (**Figure 7A**). KOG annotation assigned 18,183 genes to 25 functional categories (**Figure 7B**). The largest category was general function prediction (4,353 genes, 23.94%), followed by post-translational modification, protein turnover, and chaperones (1,998 genes, 10.99%), signal transduction mechanisms (1,742 genes, 9.58%), carbohydrate transport and metabolism (1,112 genes, 6.11%), and transcription (1,049 genes, 5.78%), which constituted the predominant functional groups. According to the GO database annotation, a total of 21,352 unigenes were classified into 52 functional categories under the three major categories of cellular component, molecular function, and biological process. Among them, the biological process category had the most entries, with 23 (**Figure 7C**). The seven entries with more than 10,000 annotated genes were: the biological process category’s cell process (14,298 entries), metabolic process (11,882 entries), and single biological process (10,482 entries); the cellular component category’s cell (17,993 entries) and cell component (17,965 entries); and the molecular function category’s binding (12,679 entries) and catalytic activity (10,951 entries). KEGG pathway analysis annotated 6,133 unigenes, which were categorized into six major pathway groups at Level 1 (**Figure 7D**). Within these, the Level 2 metabolism category encompassed sugar metabolism (1,046 entries), amino acid metabolism (597 entries), and energy metabolism (545 entries), among others. The genetic information processing category at Level 2 included prominent pathways such as translation (957 entries) and folding, sorting, and degradation (718 entries).

### 3.5 Differential gene expression analysis under drought stress

**Figure 8:**
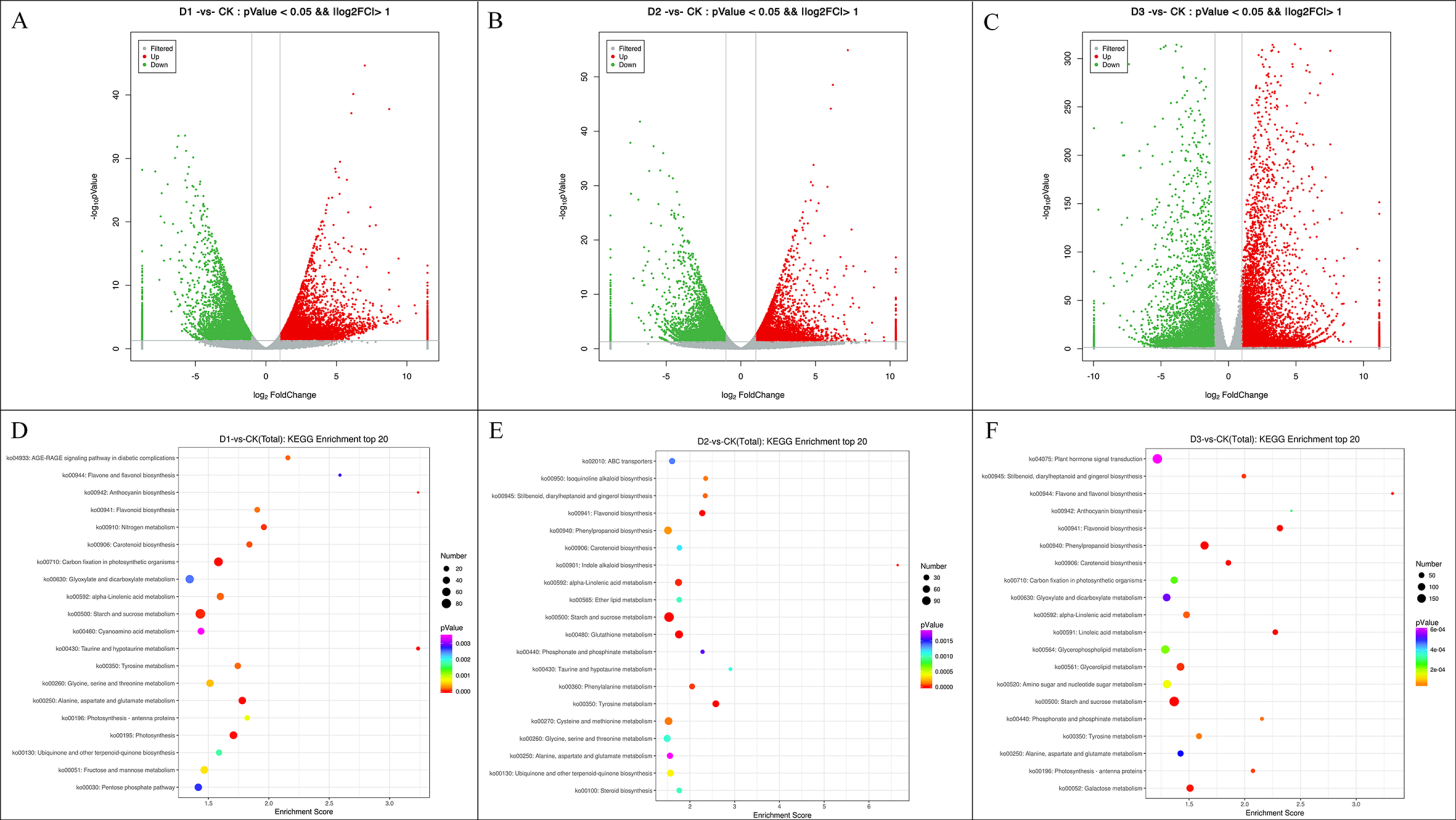
Screening of DEGs and KEGG pathway enrichment analysis. **(A–C)** Volcanic map distribution of different genes of cranberry under different degrees of drought stress. The x-axis represents log_2_ fold change (log_2_FC), where a value > 1 indicates up-regulation and a value < -1 indicates down-regulation (the core threshold for DEG screening). The y-axis represents -log_10_ (p-Value), reflecting the statistical significance of gene expression differences (higher values indicate greater significance). Red dots represent significantly up-regulated DEGs (log_2_FC > 1, FDR < 0.05), green dots represent significantly down-regulated DEGs (log_2_FC < -1, FDR < 0.05), and gray dots represent non-differentially expressed genes. **(D–F)**, KEGG pathway enrichment bubble plots of DEGs under varying drought stress conditions. The x-axis denotes the enrichment score (enrichment factor), while the y-axis lists the top 20 KEGG pathways ranked by enrichment degree. The bubble size corresponds to the number of DEGs annotated to each pathway (larger bubbles indicate a greater number of genes; smaller bubbles indicate fewer genes). The color gradient of the bubbles represents the enrichment −log_10_(p-value) (i.e., statistical significance), transitioning from blue to red to indicate that a smaller p-value corresponds to higher statistical significance of pathway enrichment.

Gene expression levels were normalized and subjected to differential expression analysis using the DESeq2 software. A negative binomial distribution test was employed to assess the statistical significance of expression differences. Differentially expressed genes (DEGs) were identified based on the criteria of |log_2_FC| > 1 and p-value < 0.05. Volcano plots were subsequently generated to visualize DEGs across sample comparisons (**Figure 8A–C**). In comparison with the control group, 15,277, 10,537, and 19,829 DEGs were identified in the D1, D2, and D3 groups, respectively. KEGG enrichment analysis was performed to identify the top 20 significantly enriched pathways, which were visualized using bubble plots (**Figure 8D–F**). **Supplementary Figure 1** presents the top 20 KEGG enrichment results for each treatment group. Among these, six KEGG metabolic pathways were consistently and significantly enriched in cranberry under all three treatment conditions, including alanine, aspartate, and glutamate metabolism; photosynthesis; starch and sucrose metabolism; carotenoid biosynthesis; flavonoid biosynthesis; and carbon fixation in photosynthetic organisms (**Supplementary Table 4**). To accurately identify core genes demonstrating co-expression across various treatment conditions while possessing definitive biological functions, this study initially employed screening criteria of |log_2_FC| > 1 and p < 0.05 to identify DEGs in each group. Following the exclusion of genes lacking explicit KEGG pathway annotations or with unknown functions, a total of 481 DEGs exhibiting co-response in all three treatment groups and annotated to KEGG pathways were obtained (**Supplementary Figure 2**). Building upon this foundation, through comparison of the characteristics of fold-change variations in gene expression under different stress gradients, core genes with stable expression patterns and consistently altered regulation across multiple treatment levels were further screened. Ultimately, 55 key genes persistently differentially expressed throughout all conditions were identified. The remaining differentially expressed genes that show inconsistent expression trends or have a relatively weak functional correlation with the core research topic are not further emphasized in the main analysis. These genes are implicated in 54 metabolic pathways (**Supplementary Table 5**). Pathways containing more than three genes included pyruvate metabolism (ko00620); carbon fixation in photosynthetic organisms (ko00710); glycine, serine, and threonine metabolism (ko00260); endocytosis (ko04144); plant MAPK signaling pathway (ko04016); and plant–pathogen interaction (ko04626).

### 3.6 DEGs associated with photosynthetic processes

**Figure 9:**
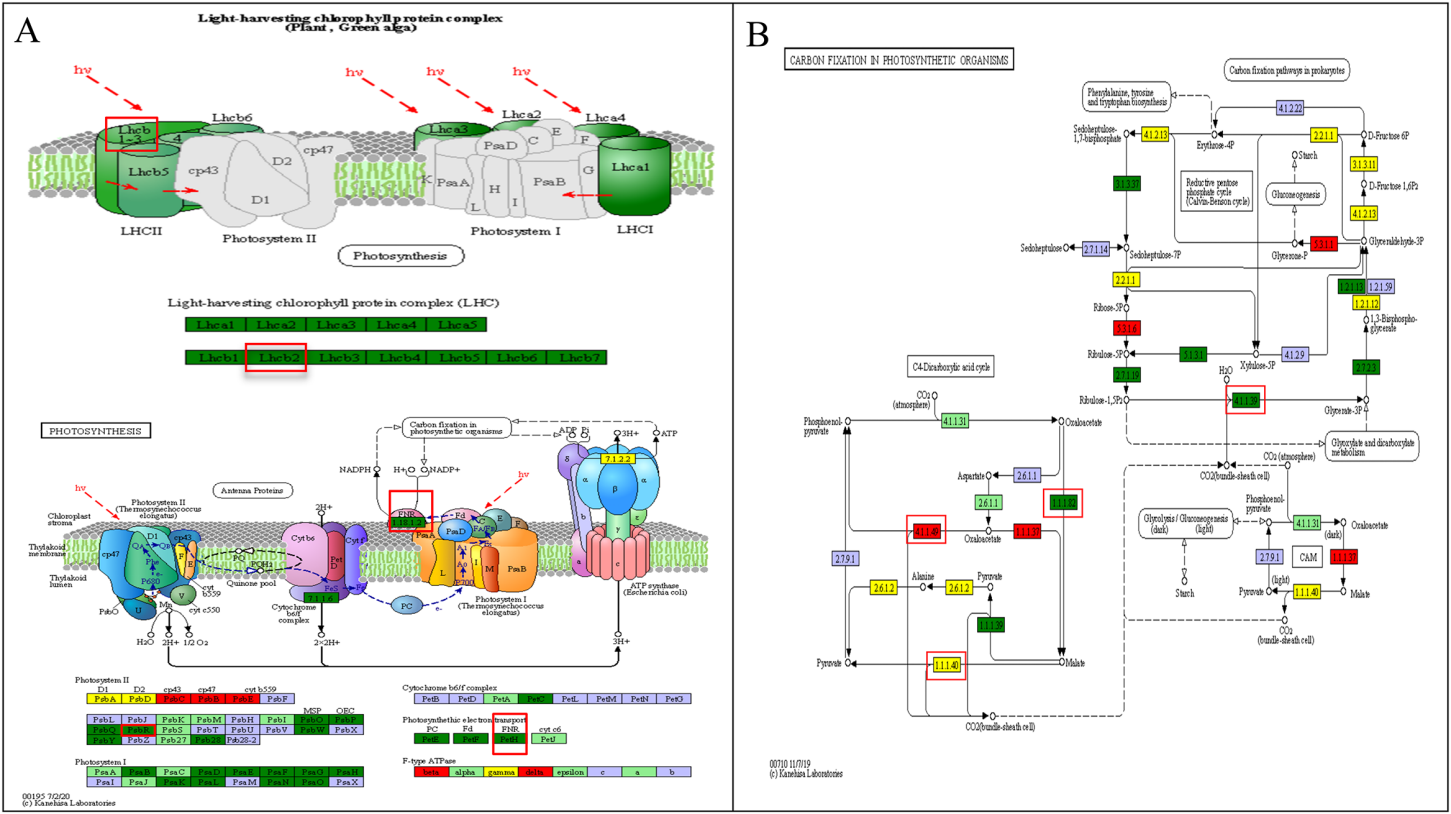
KEGG enrichment analysis of key photosynthetic pathways in cranberry. **(A)** Photosynthetic antenna protein and photosynthesis KEGG of cranberry. **(B)** KEGG of carbon assimilation pathway for photosynthesis of cranberry. Red circle is the target gene.

Analysis revealed that there were three pathways with the highest response to drought stress in terms of photosynthesis, involving the largest number of genes, totaling seven. These DEGs were respectively enriched in the photosynthesis, photosynthetic antenna protein, and carbon fixation in photosynthetic organisms pathways (**Figure 9**). Specifically, the photosynthesis pathway included two continuously DEGs, *psbR* and *petH*; the photosynthetic antenna protein pathway contained one continuously differentially expressed gene, *LHCB2* (**Figure 9A**); and the carbon fixation in photosynthetic organisms pathway covered four continuously DEGs, *E4.1.1.49* (pckA), *E1.1.1.82*, *E1.1.1.40* (maeB), and *rbcS* (**Figure 9B**).

**Figure 10:**
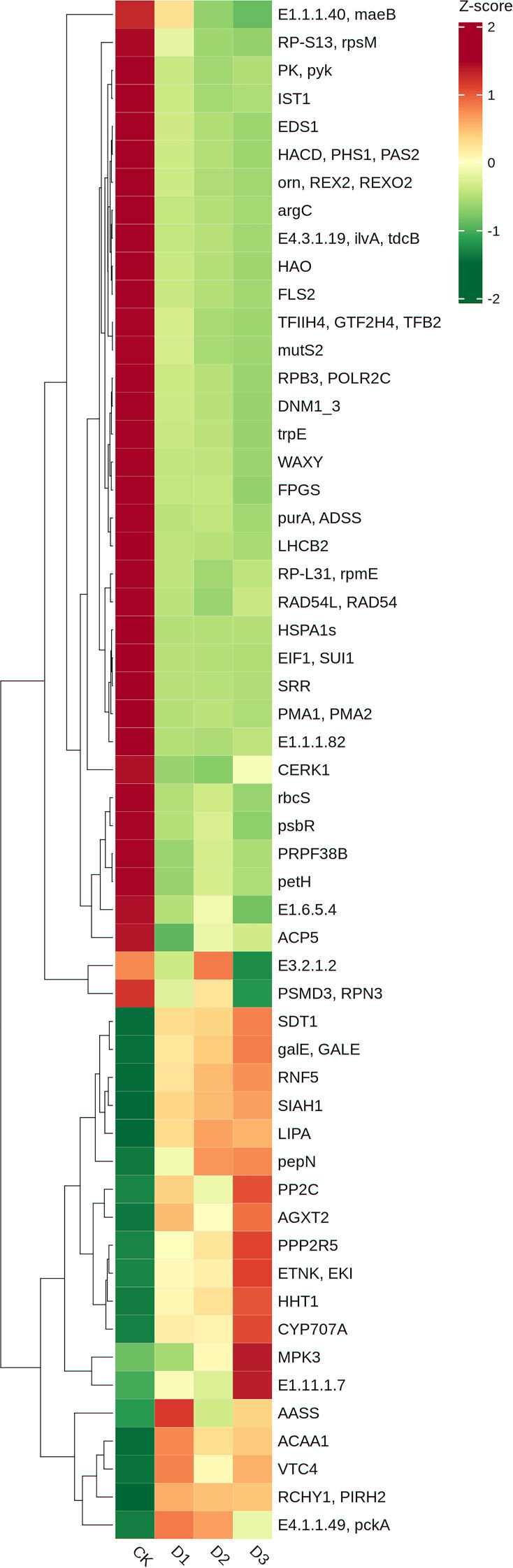
Different gene expressions of cranberry under drought stress. The rows represent individual genes, and the columns represent the control (CK) and drought stress treatments (D1, D2, D3). The DEGs were screened using the thresholds of |log_2_ fold change (|log_2_FC)| > 1 and FDR < 0.05. The color scale on the right indicates the Z-score normalized gene expression values: red represents high relative expression (Z-score > 0), while green represents low relative expression (Z-score < 0). Genes were hierarchically clustered based on their expression profiles across all samples to show co-expression patterns under drought stress.

Based on the KEGG annotation information (**Supplementary Table 5**), the functions of each gene are as follows: *LHCB2* encodes the chlorophyll a/b binding protein 2 of light-harvesting complex II, belonging to the LHCIIb subclass, and is a major component of the light-harvesting pigment-protein trimer in higher plants (**Figure 9A**); *psbR* encodes the 10-kDa protein of photosystem II, which is predicted to be located near the oxygen-evolving complex (OEC) of photosystem II, but this prediction has not been experimentally verified, and thus is not annotated in the KEGG pathway map (**Figure 9A**); *petH* encodes ferredoxin-NADP^+^ reductase (FNR), located at the end of photosystem I, and is a key terminal enzyme in the photosynthetic electron transport chain; *E4.1.1.49 (pckA)* encodes phosphoenolpyruvate carboxykinase, which participates in photosynthetic carbon fixation through decarboxylation, providing a carbon source for gluconeogenesis, maintaining pH balance, and regulating stomatal aperture, and are at the metabolic intersection of glycolysis/gluconeogenesis and the tricarboxylic acid (TCA) cycle (**Supplementary Table 5**); *E1.1.1.82* encodes NADP^+^-dependent malate dehydrogenase, involved in malate and pyruvate metabolism; *E1.1.1.40 (maeB)* encodes oxaloacetate decarboxylase, regulating the decarboxylation of oxaloacetate and pyruvate metabolism during photosynthetic carbon assimilation; *rbcS* encodes ribulose-1,5-bisphosphate carboxylase (RuBisco), the core enzyme of plant carbon assimilation, directly affecting photosynthetic efficiency. In terms of expression patterns, only *E4.1.1.49 (pckA)* were continuously upregulated under escalating drought stress gradients, while the other six genes, *psbR, petH, LHCB2, E1.1.1.82, E1.1.1.40 (maeB)*, and *rbcS*, were continuously downregulated (**Figure 10**).

**Figure 11:**
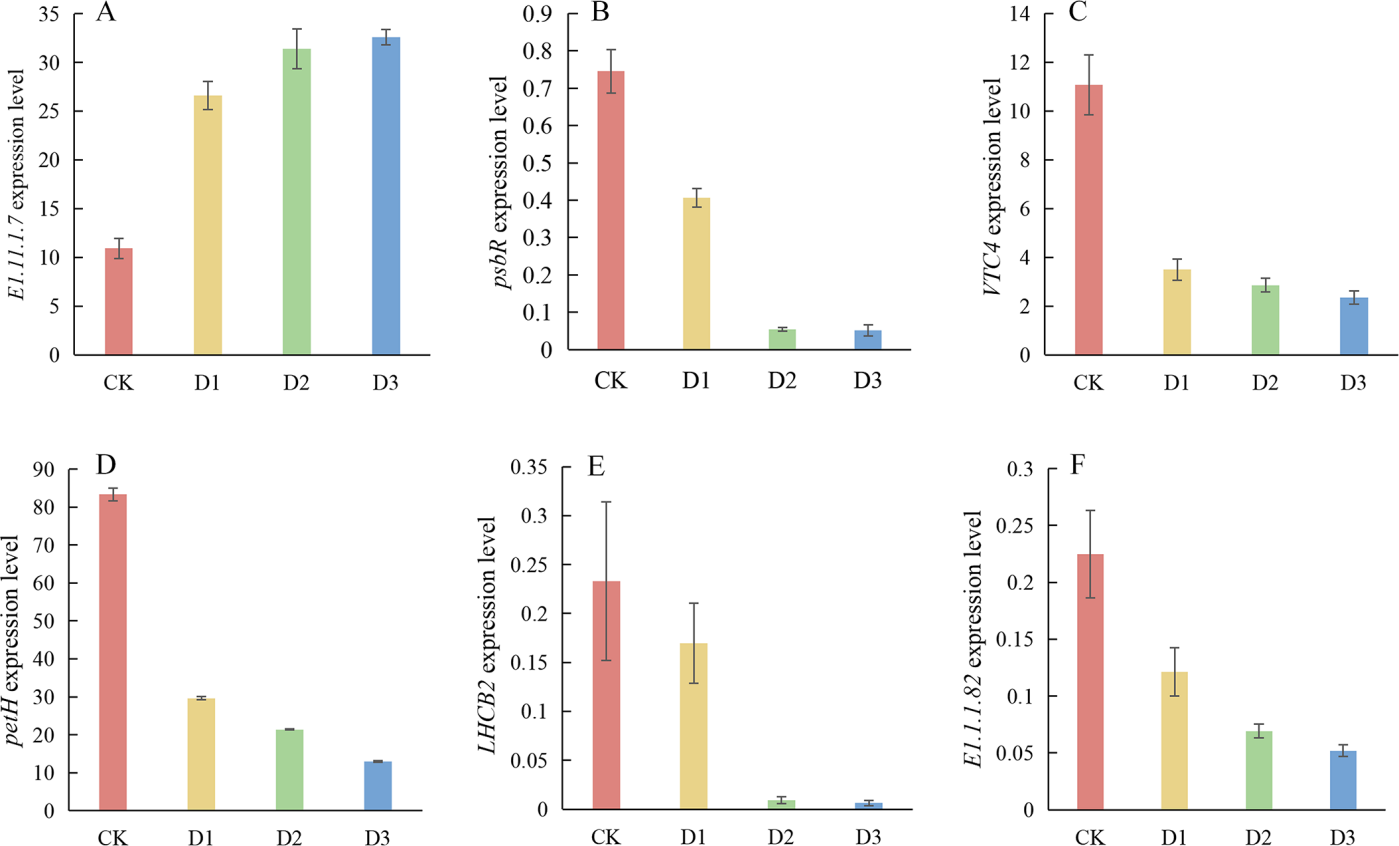
Quantitative results of qRT-PCR differential genes. **(A)** *E1.11.1.7*; **(B)** *psbR*; **(C)** *VTC4*; **(D)** *petH*; **(E)** *LHCB2*; **(F)**, *E1.1.1.82.*.

To assess the accuracy of transcriptome sequencing, this study randomly selected six genes that consistently responded to drought stress for qRT-PCR analysis. The selected genes include *LHCB2* (light-harvesting complex chlorophyll a/b binding protein 2), *E1.11.1.7* (peroxidase), *petH* (ferredoxin-NADP^+^ reductase), *psbR* (Photosystem II 10 kDa protein), *VTC4* (inositol phosphatase/L-galactose 1-phosphatase), and *E1.1.1.82* (malate dehydrogenase). The quantitative results of qRT-PCR are presented in **Figure 11**. Among the six genes analyzed, *E1.11.1.7* exhibited upregulation in expression, whereas the other five genes demonstrated downregulation. The down-regulation pattern of photosynthesis-related genes verified by qRT-PCR was consistent with the transcriptome data (**Figure 11**), and the changes in the expression levels of these genes corresponded to the physiological indicators such as the decline in chlorophyll content, the reduction in PSII photochemical efficiency, and the increase in reactive oxygen species levels under the stress gradient.

## 4 Discussion

### 4.1 Responses of photosynthetic physiology of cranberries under drought stress

Drought stress, a major abiotic constraint on plant growth, significantly impairs photosynthetic performance by reducing the net photosynthetic rate. In this study, the photosynthetic oxygen evolution in cranberry leaves exhibited a progressive decline with increasing drought stress. The decline in the plant photosynthetic rate can be broadly classified into stomatal limitation and non-stomatal limitation (Gao et al., 2019; Shi et al., 2020; Nadal-Sala et al., 2021). However, owing to the diminutive size of cranberry leaves, precise quantification of intercellular CO2 concentration and stomatal conductance via standard infrared gas exchange systems was technically unfeasible; consequently, we could not definitively partition the observed photosynthetic decline into stomatal versus non-stomatal components. Notwithstanding this limitation, the leaf anatomical results indicated that drought stress significantly reduced the stomatal density and stomatal aperture ratio of cranberry leaves. Under water deficit, stomatal closure is often the first and most sensitive response limiting CO_2_ diffusion into the leaf, thereby reducing transpiration and conserving water while also lowering CO_2_ assimilation (Chaves, 1991). Under mild to moderate drought conditions, the decline in photosynthetic rates is primarily attributable to stomatal limitation. Consequently, the observed reduction in stomatal aperture in this study likely became the principal factor constraining photosynthesis by impeding intercellular CO_2_ concentration. In contrast, stomatal density is developmentally regulated and further modulated by leaf ontogeny, phyllotactic position, and senescence-related processes (Zhang et al., 2025). Consistent with our observations, Xu et al. reported that severe drought stress significantly reduces stomatal density in plant leaves (Xu and Zhou, 2008). However, recent evidence suggests that stomatal density may increase under water deficit in certain species (Kang et al., 2025). These inconsistencies may be attributed to species-specific adaptive strategies and differences in the nature of abiotic stress conditions (Chua and Lau, 2024). Given that all sampled leaves were mature leaves and collected from consistent anatomical positions, we hypothesize that drought stress may have induced premature senescence or apoptosis of guard cells, thereby contributing to a decline in stomatal numbers.

In addition to impairing stomatal function, drought stress disrupts carbon metabolism, thereby impeding the conversion of absorbed light energy into chemical energy. OJIP curve analysis demonstrated that the relative fluorescence values at the J - and K - steps of cranberry leaves exhibited a significant increase on the 6th and 9th days of drought stress. The elevation at the J - step suggested that the electron transport on the acceptor side of PSII (from QA to QB) was impeded, whereas the rise at the K - step indicated damage to the OEC on the donor side of PSII, the higher the K value, the more pronounced the damage to the donor-side OEC of PSII. Collectively, these findings suggested that both D2 and D3 treatments caused damage to the donor side of PSII in cranberry, with more severe impairment observed in D3 treatment. Similar findings have been reported in studies on blue honeysuckle (Yan et al., 2024) and sorghum (Zhang et al., 2019). Mechanistically, the potential mechanisms underlying the damage to the OEC on the donor side of PSII may involve the overaccumulation of excitation energy due to impaired electron transfer on the acceptor side, as well as the inhibition of the PSII water-splitting system resulting from plant water deficit (Kono et al., 2022). Furthermore, the relative fluorescence intensity at the L-band gradually increased with the progression of drought stress. The L-point serves as an indicator of energy transfer connectivity among PSII components, where elevated values reflect reduced connectivity (Wang et al., 2018). Thus, the functional connectivity among PSII components in cranberry leaves declined under increasing drought severity. Chlorophyll fluorescence parameters provide valuable insights into photosynthetic electron transport efficiency and thermal dissipation processes during photosynthesis (Baker, 2008). PCA revealed that the PSII performance of cranberry was lower than that of CK under D2 conditions, concomitant with a marked enhancement in its heat dissipation capacity In contrast,. under D3 conditions, PSII functionality was severely compromised, as evidenced by the lowest recorded values of multiple fluorescence-derived parameters—including Fv’/Fm’, qP, Rfd, and ΦPSII—alongside a pronounced decline in NPQ compared to D2. Consistent with these observations, chlorophyll fluorescence transient analysis (measured using an FMS-2 fluorometer) revealed elevated fluorescence intensities at the K- and J-steps under D3, indicative of impaired electron transfer beyond QA− and accumulation of excess excitation energy within the photosynthetic electron transport chain. Collectively, these results suggest that under D2, cranberry leaves sustain PSII integrity primarily through downregulation of light harvesting (e.g., reaction center closure and reduced excitation capture efficiency) and enhanced thermal dissipation; whereas under D3, the collapse of both photochemical efficiency and NPQ capacity reflects irreversible damage to the PSII apparatus and failure of photoprotective mechanisms. Consistent with the PSII functional deterioration observed under D3, ultrastructural analysis of mesophyll cells revealed pronounced thylakoid swelling, distortion of grana lamellae, and eventual disintegration of chloroplast membranes. This structural disassembly of the photosynthetic apparatus provides a direct mechanistic explanation for the irreversible loss of PSII photochemical efficiency and the failure of thermal dissipation (NPQ), thereby linking drought-induced chloroplast ultrastructure damage to the collapse of photosynthetic function.

### 4.2 Responses of leaf anatomy in cranberry under drought stress

As the plant organ most vulnerable to drought stress, leaves undergo structural alterations that serve as critical indicators for assessing drought resistance (Liu et al., 2023). This study demonstrates that under mild drought conditions, palisade tissue thickness increases, whereas the upper and lower epidermis and spongy mesophyll exhibit reduced thickness, although these changes are not statistically significant. However, with prolonged exposure and increasing intensity of drought stress, the thickness of the palisade tissue, spongy mesophyll, and both upper and lower epidermis decreases significantly. Under severe stress, the cell layers of the palisade and spongy mesophyll become markedly thinner, with cells displaying disorganized and loosely packed arrangements and enlarged intercellular spaces—morphological features indicative of incipient cellular rupture. These observations are consistent with findings reported by Khan (Khan et al., 2023) and Velikova (Velikova et al., 2020), further supporting the physiological mechanism by which cellular water loss leads to reduced leaf thickness under intensified drought stress. This response pattern contrasts sharply with the adaptive strategies observed in drought-tolerant species such as jujube and olive, which enhance drought resilience through thickening of the palisade tissue, an increased palisade-to-spongy tissue ratio, formation of denser mesophyll, development of thicker cuticles, and reinforcement of vascular bundles in major veins—collectively establishing an efficient water conservation system that elevates the physiological threshold for wilting and enables survival under extreme drought conditions. The present results clearly indicate that cranberry is inherently sensitive to drought stress. In the early stages, the transient thickening of the palisade tissue may represent a short-term physiological adjustment to adverse conditions. Concurrently, the plant activates a conservative defense strategy involving upregulation of the enzymatic antioxidant system, including enhanced activities of SOD and CAT, to maintain cellular redox homeostasis. At this stage, the membrane system remains largely intact, consistent with the preservation of mesophyll ultrastructure.

However, under moderate to severe drought stress, the rate of ROS production exceeds the scavenging capacity of the antioxidant system, resulting in a sharp accumulation of MDA and H_2_O_2_, which reach peak levels under D2 and D3 stress conditions, respectively. This pattern of oxidative damage aligns with responses documented in UCB-1 pistachio rootstocks (Pakzad et al., 2019) and licorice (Hosseini et al., 2018). Uncontrolled oxidative stress directly induces ultrastructural damage in plant cells. Chloroplasts, as primary sites of photosynthesis and ROS generation, are particularly susceptible to stress-induced injury. By integrating the dynamic changes of ROS and antioxidant enzyme activities, we found that under D1 conditions, the activities of SOD and CAT increased in tandem with the moderate rise of H_2_O_2_ and MDA, which corresponded to the basic integrity of chloroplast ultrastructure. However, under D2 and D3 conditions, the further increase in ROS generation exceeded the scavenging capacity of SOD and CAT, coinciding with the sharp accumulation of MDA and H_2_O_2_. This imbalance directly led to progressive damage to chloroplast ultrastructure, from thylakoid swelling to complete chloroplast disintegration, indicating that the failure of antioxidant defense at the transcriptional and enzymatic levels initiated and exacerbated oxidative damage to organelle membranes. Initial signs of chloroplast degradation include a marked reduction in starch grain accumulation and an increase in osmiophilic granules, progressing to thylakoid swelling, distortion, disintegration, and breakdown of stroma lamellae—ultrastructural alterations consistent with previously reported damage to thylakoid membranes (Atkin and Macherel, 2009; Anjum et al., 2011). Under conditions of severe drought, the grana undergo complete disintegration and chloroplast structure collapses entirely, resulting in compromised membrane integrity and loss of photosynthetic function. Concurrently, rupture of mitochondrial membranes and disintegration of the nuclear envelope occur, indicating the activation of programmed cell death (Daneva et al., 2016). Ultimately, extensive degradation of chloroplast and mitochondrial membranes leads to irreversible cessation of photosynthesis and respiration, accompanied by cellular dehydration and structural breakdown. Although our experiments have clarified the dynamic changes in the ultrastructure of chloroplasts and osmium acid affinity particles under drought stress, further studies are still needed to verify and quantify the statistical patterns of the dynamic changes in chloroplast ultrastructure and osmium acid affinity particles through quantitative analysis and continuous section observation, so as to provide more accurate evidence support for the response mechanism of plant photosynthetic structure under drought stress.

### 4.3 Responses of DEGs in photosynthesis

Transcriptome analysis has been widely employed to investigate plant responses to drought stress at the transcriptional level, enabling the regulation of signaling pathways and physiological processes that facilitate adaptation to water-deficient conditions (Yang et al., 2014). In the present study, a relatively large number of DEGs were detected under drought stress, particularly in the D1 and D3 treatments. This likely reflects comprehensive transcriptional reprogramming in cranberry plants in response to progressively intensifying water deficit. Similar phenomena have been reported in other crops; for instance, in quinoa (Chenopodium quinoa), drought stress also induced large-scale transcriptome remodeling, with over 10,000 DEGs identified, involving key pathways such as starch and sucrose metabolism (Huan et al., 2022). Given that cranberry is a non-model species lacking a chromosome-level reference genome, transcriptome analysis relies on *de novo* assembly. Although necessary for non-model organisms, studies have shown that the *de novo* assembly process is complex and susceptible to generating fragmented or chimeric transcripts, which can increase transcript redundancy and potentially affect the absolute number of identified DEGs (Huang et al., 2016). Moreover, intrinsic biological variation and systemic activation of general stress response pathways may also contribute to the observed relatively high number of DEGs (Li et al., 2025). Despite the high total number of DEGs, through in-depth functional annotation and expression pattern clustering, we successfully identified 55 core genes exhibiting sustained differential expression, including 19 consistently up-regulated and 36 consistently down-regulated genes. These genes were primarily involved in pyruvate metabolism (ko00620), carbon fixation in photosynthetic organisms (ko00710), glycine, serine, and threonine metabolism (ko00260), endocytosis (ko04144), plant MAPK signaling pathway (ko04016), and plant-pathogen interaction (ko04626). Notably, among the consistently DEGs, those associated with photosynthesis represented the highest proportion, accounting for 13%. The *LHCB2* gene, which encodes a constituent of LHCBII, is involved in the Photosynthesis-antenna proteins (ko00196) pathway. LHCB2 functions as a peripheral antenna associated with the PSII reaction center and is commonly recognized as a light-harvesting chlorophyll a/b-binding protein. Its primary role involves capturing light energy and transferring it to the PSII reaction center (Green and Durnford, 1996). Under excessive excitation energy conditions in PSII, LHCII can be phosphorylated, leading to its dissociation from PSII and subsequent migration to PSI. Conversely, when PSI receives excess excitation energy, increased protein phosphatase activity promotes dephosphorylation of LHCII, enabling its reassociation with PSII. This reversible phosphorylation mechanism dynamically modulates the light-harvesting capacity of the two photosystems, thereby balancing the distribution of excitation energy between them. In the present study, qPCR results showed that LHCB2 expression was progressively down-regulated with increasing intensity of drought stress, consistent with the transcriptome sequencing data. This finding suggests that under initial drought conditions, cranberry down-regulates LHCB2 expression to reduce chlorophyll content and attenuate light-harvesting capacity, thus optimizing excitation energy distribution within the photosynthetic apparatus. Supporting this interpretation, previous studies have demonstrated that Arabidopsis thaliana mutants with suppressed LHCB2 expression exhibited reduced chlorophyll content, leaf bleaching, diminished light-harvesting efficiency, and impaired capacity for NPQ (Jenny et al., 2003). However, as drought stress persists and exceeds a critical threshold, the heat dissipation capacity of cranberry leaves becomes compromised, resulting in a significant decline in PSII photochemical efficiency and NPQ. The sustained down-regulation of LHCB2 expression under increasing drought intensity paralleled the observed reduction in chlorophyll content and the diminished NPQ capacity under D3 conditions, indicating that transcriptional suppression of light-harvesting antenna proteins underlies the compromised photoprotective thermal dissipation.

In addition, the *psbR* and *petH* genes were identified to be associated with the photosynthesis (ko00195) pathway. PsbR interacts with PsbP and PsbQ to stabilize the donor side of PSII and ensure its structural integrity and functional competence. Evidence from prior studies indicates that PsbR deficiency markedly impairs light-saturated oxygen evolution and reduces PSII activity in plants (Allahverdiyeva et al., 2013). In the present study integrating the expression profile of *psbR* under severe drought stress, we infer that the observed decline in photosynthetic oxygen evolution, structural damage to the PSII donor side, and compromised PSII performance in cranberry are attributable to pronounced down-regulation of *psbR*. This transcriptional pattern is further corroborated by the ultrastructural evidence showing thylakoid swelling and disintegration of the oxygen-evolving complex (OEC)-associated membranes under severe drought, thereby establishing a direct link between psbR down-regulation, structural disassembly of the PSII donor side, and the functional loss of oxygen evolution capacity. The *petH* gene encodes FNR, which exists in two major isoforms: leaf-type FNR (LFNR) and root-type FNR (RFNR). LFNR is predominantly expressed in photosynthetic tissues, where it catalyzes the final step of the photosynthetic electron transport chain—reduction of NADP^+^ to NADPH—for subsequent use in the Calvin cycle. In contrast, RFNR is expressed in non-photosynthetic tissues and participates in diverse metabolic processes (Grabsztunowicz et al., 2021). Our transcriptomic analysis revealed that *petH* expression was consistently down-regulated with increasing drought stress severity. Concurrently, H_2_O_2_ levels in cranberry leaves increased proportionally with drought intensity. Previous studies have shown that oxidative stress can suppress the expression of FNR-encoding genes and trigger dissociation of FNR from thylakoid membranes—a regulatory response proposed to maintain redox homeostasis of the NADP^+^/NADPH pool (Palatnik et al., 1997). FNR released from the thylakoid membrane is known to associate with the Cytb6f complex and participate in the water-to-water cycle, thereby mitigating photodamage to PSI under excess excitation energy (Lehtimäki et al., 2010). Collectively, our observations indicate that drought-induced down-regulation of *petH* serves a protective function: by modulating NADP^+^/NADPH redox balance, it helps alleviate PSI photoinhibition and partially preserves carbon assimilation capacity under stress conditions.

Ultimately, the genes *rbcS*, *E1.1.1.82*, *E1.1.1.40* (maeB), and *E4.1.1.49* (pckA) were found to be associated with the Carbon fixation in photosynthetic organisms (ko00710) pathway. Among these genes, *rbcS* is one of the most strongly light-inducible genes, encoding the small subunit of RuBisCo in higher plants. When plants are exposed to adverse stress conditions, the large subunit of RuBisCo tends to dissociate, leading to a reduction in both the activity and content of RuBisCo. The expression level of *rbcL*, which encodes the large subunit of RuBisCo, is regulated by *rbcS*; therefore, *rbcS* plays a critical role in determining the efficiency of plant photosynthesis (Mi et al., 2018). In cranberries, our data show that progressive drought stress induces a marked downregulation of rbcS expression, which likely contributes to diminished carbon assimilation capacity and concomitant reduction in photosynthetic oxygen evolution. Additionally, AGXT2, a gene associated with the alanine, aspartate, and glutamate metabolism pathway (ko00250), exhibited a progressive upregulation under intensified drought stress. These expression changes of the aforementioned genes reflect the adaptive responses of cranberries to drought stress, which may help the plant cope with the adverse effects of water deficit on physiological metabolism.

## 5 Conclusions

This study reveals the phased response strategies of cranberry in coping with drought stress. Under mild drought, the plants mainly exhibit tolerance by maintaining the stability of the photosynthetic system. Under moderate drought, photosynthesis is limited by both stomatal and non-stomatal factors, but cranberry initiates a self-protection mechanism centered on enhancing non-photochemical quenching (NPQ) and the activities of antioxidant enzymes (SOD, CAT), effectively alleviating oxidative damage. However, when drought intensifies to severe levels, the protective mechanism fails, non-stomatal limitations become dominant, leading to irreversible damage to the oxygen-evolving complex (OEC) and disintegration of the thylakoid membrane in chloroplasts, causing the collapse of the photosynthetic apparatus and an imbalance in reactive oxygen species metabolism. Transcriptome analysis further confirmed this physiological process at the molecular level. The coordinated down-regulation of genes related to photosynthesis pathways (such as LHCB2, psbR, rbcS, petH) is the direct molecular cause of the decline in photosynthetic efficiency. The 55 differentially expressed genes identified in this study that continuously respond to drought, especially those related to photosynthesis, provide key targets for understanding the molecular network of cranberry drought resistance. These findings not only deepen the understanding of the drought response mechanism of cranberry but also lay a solid theoretical foundation for future targeted improvement of cranberry drought resistance through molecular marker-assisted breeding or cultivation regulation.

## Data Availability

The original contributions presented in the study are included in the article/**Supplementary Material**. Further inquiries can be directed to the corresponding authors.

## Supplementary material

Supplementary Table 1 Primers used for qRT-PCR.

Supplementary Table 2 Base information statistics before and after transcriptome filtering of cranberry under drought stress.

Supplementary Table 3 Unigenes results of functional annotations.

Supplementary Table 4 Number of KEGG pathways and their differentially expressed genes significantly enriched in cranberry under different degrees of drought stress.

Supplementary Table 5 Genes with persistent expression in cranberry under varying degrees of drought stress.

Supplementary Figure 1 Venn diagram of common KEGG enriched pathways of differentially expressed genes in cranberry under different drought stress treatments

Supplementary Figure 2 Common differentially expressed genes of cranberry in response to KEGG pathway under different drought stress.
