# Supplementary material for: Physiological and transcriptomic analysis of cranberry (Vaccinium macrocarpon) in response to drought stress
Source: Front Plant Sci. 2026 May 7;17:1797317. doi: 10.3389/fpls.2026.1797317 (PMC13189740; doi:10.3389/fpls.2026.1797317)
Supplement: Supplementary Table 1 — Primers used for qRT-PCR. [file Table1.docx]

**Table S1. Primers used for qRT-PCR.**

| Gene number | KEGG name | Primer sequence(5'to3') |
| --- | --- | --- |
| TRINITY_DN14022_c0_g1_i6_5 | E1.11.1.7 | F: GAGGCTCCACGGATTCTCAA  R: CACCATTGCACTGGCAAAAT |
| TRINITY_DN14751_c0_g1_i1_3 | ACAA1 | F:CGCGAGATTGCCTTCTTCCT  R:GTCCTGCTCTTGCCTCGTAACA |
| TRINITY_DN15588_c0_g2_i1_2 | E1.1.1.82 | F:GAACTTTGGATGGCCCCTTGT  R:CAACCATTGTGAACGGTGAGAA |
| TRINITY_DN16465_c0_g1_i3_8 | psbR | F:GCACTCTCTCGCACCTATTGGT  R:CGATTATAGTGTCACTCGGTGCAT |
| TRINITY_DN17731_c0_g1_i2_8 | petH | F:CTGATGGAGAGGTCCCATATAGAGA  R:GTGAGGCTTCCCATTCTTGTCA |
| TRINITY_DN21227_c0_g1_i1_3 | rbcS | F:ACCCGCAAGACCAACACTGA  R:AGTGGAGGCCATACCTGCAT |
| TRINITY_DN10019_c0_g1_i1_2 | ATPF1D, atpH | F:AACAAACCCCGATCACATTGC  R:GGTGGTTCTGTAAGCGTGGAA |
| TRINITY_DN14080_c0_g1_i7_6 | petJ | F:TGGTTTTCCCCTTCTCTTTCG  R:CGAAAGCAAGGAGCCAATTC |
| TRINITY_DN16108_c0_g1_i1_1 | psbP | F:TCGGCTTGGAACGTATAGGAA  R:TTGGAGCAGCGGATATTTGC |
| TRINITY_DN16310_c0_g1_i7_6 | psbB | F:GGCGCGAACTTTTTGTACGT  R:AACAATTCCGTCGCCGTCTA |
| TRINITY_DN22612_c0_g1_i1_5 | psbA | F:TGTATTCGGCGGCTCTCTATTC  R:CATTTTCGGTGGTTTCCCTGAT |
