# Supplementary material for: Physiological and transcriptomic analysis of cranberry (Vaccinium macrocarpon) in response to drought stress
Source: Front Plant Sci. 2026 May 7;17:1797317. doi: 10.3389/fpls.2026.1797317 (PMC13189740; doi:10.3389/fpls.2026.1797317)
Supplement: Supplementary Table 2 — Base information statistics before and after transcriptome filtering of cranberry under drought stress. [file Table2.docx]

**Table S2. Base information statistics before and after transcriptome filtering of cranberry under drought stress.**

| Treatment | Raw Reads | Raw Bases | Clean Reads | Clean Bases | Q3（%） | GC（%） |
| --- | --- | --- | --- | --- | --- | --- |
| CK_1 | 47.59M | 7.14G | 47.20M | 6.85G | 95.26% | 46.03% |
| CK_2 | 51.07M | 7.66G | 50.63M | 7.33G | 95.12% | 46.14% |
| CK_3 | 50.51M | 7.58G | 50.10M | 7.26G | 95.16% | 46.12% |
| D1_1 | 49.00M | 7.35G | 48.60M | 7.02G | 95.55% | 46.32% |
| D1_2 | 43.19M | 6.48G | 42.86M | 6.23G | 95.14% | 46.50% |
| D1_3 | 47.44M | 7.12G | 47.06M | 6.81G | 95.38% | 46.37% |
| D2_1 | 51.26M | 7.69G | 50.85M | 7.33G | 95.23% | 45.91% |
| D2_2 | 47.22M | 7.08G | 46.82M | 6.74G | 95.48% | 46.31% |
| D2_3 | 51.52M | 7.73G | 51.08M | 7.36G | 95.48% | 46.54% |
| D3_1 | 49.12M | 7.37G | 48.67M | 7.06G | 95.17% | 46.39% |
| D3_2 | 48.17M | 7.23G | 47.77M | 6.91G | 95.37% | 46.56% |
| D3_3 | 44.61M | 6.69G | 44.23M | 6.38G | 95.34% | 46.66% |
