# Supplementary material for: Physiological and transcriptomic analysis of cranberry (Vaccinium macrocarpon) in response to drought stress
Source: Front Plant Sci. 2026 May 7;17:1797317. doi: 10.3389/fpls.2026.1797317 (PMC13189740; doi:10.3389/fpls.2026.1797317)
Supplement: Supplementary Table 3 — Unigenes results of functional annotations. [file Table3.docx]

**Table S3. Unigenes results of functional annotations.**

| Database | The number of genes annotatedinto the database | As a percentage of totalnumber of genes (%) |
| --- | --- | --- |
| NR | 30910 | 53.27 % |
| KOG | 18183 | 31.34 % |
| G0 | 21352 | 36.80 % |
| KEGG | 6133 | 53.27 % |
