# Supplementary material for: Physiological and transcriptomic analysis of cranberry (Vaccinium macrocarpon) in response to drought stress
Source: Front Plant Sci. 2026 May 7;17:1797317. doi: 10.3389/fpls.2026.1797317 (PMC13189740; doi:10.3389/fpls.2026.1797317)
Supplement: Supplementary Table 4 — Number of KEGG pathways and their differentially expressed genes significantly enriched in cranberry under different degrees of drought stress. [file Table4.docx]

**Table S4. Number of KEGG pathways and their differentially expressed genes significantly enriched in cranberry under different degrees of drought stress.**

| Pathway definition | Pathway | D1-CK | | D2-CK | | D3-CK | |
| --- | --- | --- | --- | --- | --- | --- | --- |
|  |  | Down | Up | Down | Up | Down | Up |
| Alanine, aspartate and glutamate metabolism | ko00250 | 15 | 29 | 10 | 20 | 18 | 26 |
| Photosynthesis | ko00195 | 34 | 14 | 24 | 7 | 45 | 9 |
| Starch and sucrose metabolism | ko00500 | 52 | 31 | 41 | 20 | 62 | 29 |
| Carotenoid biosynthesis | ko00906 | 13 | 12 | 9 | 11 | 15 | 13 |
| Flavonoid biosynthesis | ko00941 | 14 | 6 | 11 | 3 | 23 | 5 |
| Carbon fixation in photosynthetic organisms | ko00710 | 45 | 22 | 32 | 12 | 55 | 27 |
