# Supplementary material for: Physiological and transcriptomic analysis of cranberry (Vaccinium macrocarpon) in response to drought stress
Source: Front Plant Sci. 2026 May 7;17:1797317. doi: 10.3389/fpls.2026.1797317 (PMC13189740; doi:10.3389/fpls.2026.1797317)
Supplement: Supplementary Table 5 — Genes with persistent expression in cranberry under varying degrees of drought stress. [file Table5.docx]

**Table S5. Genes with persistent expression in cranberry under varying degrees of drought stress**

|  | Pathway | Pathway definition | KEGG gene name | KEGG description |
| --- | --- | --- | --- | --- |
| 1 | ko00010 | Glycolysis | E4.1.1.49, pckA | phosphoenolpyruvate carboxykinase (ATP) [EC:4.1.1.49] |
|  |  |  | PK, pyk | pyruvate kinase [EC:2.7.1.40] |
| 2 | ko00020 | Gluconeogenesis\|Citrate cycle (TCA cycle) | E4.1.1.49, pckA | phosphoenolpyruvate carboxykinase (ATP) [EC:4.1.1.49] |
| 3 | ko00620 | Pyruvate metabolism | E4.1.1.49, pckA | phosphoenolpyruvate carboxykinase (ATP) [EC:4.1.1.49] |
|  |  |  | E1.1.1.82 | malate dehydrogenase (NADP+) [EC:1.1.1.82] |
|  |  |  | PK, pyk | pyruvate kinase [EC:2.7.1.40] |
|  |  |  | E1.1.1.40, maeB | malate dehydrogenase (oxaloacetate-decarboxylating)(NADP+) [EC:1.1.1.40] |
| 4 | ko00710 | Carbon fixation in photosynthetic organisms | E4.1.1.49, pckA | phosphoenolpyruvate carboxykinase (ATP) [EC:4.1.1.49] |
|  |  |  | E1.1.1.82 | malate dehydrogenase (NADP+) [EC:1.1.1.82] |
|  |  |  | E1.1.1.40, maeB | malate dehydrogenase (oxaloacetate-decarboxylating)(NADP+) [EC:1.1.1.40] |
|  |  |  | rbcS | ribulose-bisphosphate carboxylase small chain [EC:4.1.1.39] |
| 5 | ko00230 | Purine metabolism | purA, ADSS | adenylosuccinate synthase [EC:6.3.4.4] |
|  |  |  | PK, pyk | pyruvate kinase [EC:2.7.1.40] |
| 6 | ko00052 | Galactose metabolism | galE, GALE | UDP-glucose 4-epimerase [EC:5.1.3.2] |
| 7 | ko00520 | Galactose metabolism\|Amino sugar and nucleotide sugar metabolism | galE, GALE | UDP-glucose 4-epimerase [EC:5.1.3.2] |

continued table:

|  | Pathway | Pathway definition | KEGG gene name | KEGG description |
| --- | --- | --- | --- | --- |
| 8 | ko00053 | Ascorbate and aldarate metabolism | E1.6.5.4 | monodehydroascorbate reductase (NADH) [EC:1.6.5.4] |
|  |  |  | VTC4 | inositol-phosphate phosphatase |
| 9 | ko00562 | Inositol phosphate metabolism | VTC4 | inositol-phosphate phosphatase / L-galactose 1-phosphate phosphatase [EC:3.1.3.25 3.1.3.93] |
| 10 | ko04070 | Phosphatidylinositol signaling system | VTC4 | inositol-phosphate phosphatase / L-galactose 1-phosphate phosphatase [EC:3.1.3.25 3.1.3.93] |
| 11 | ko00062 | Fatty acid elongation | HACD, PHS1, PAS2 | very-long-chain (3R)-3-hydroxyacyl-CoA dehydratase [EC:4.2.1.134] |
| 12 | ko01040 | Biosynthesis of unsaturated fatty acids | ACAA1 | acetyl-CoA acyltransferase 1 [EC:2.3.1.16] |
|  |  |  | HACD, PHS1, PAS2 | very-long-chain (3R)-3-hydroxyacyl-CoA dehydratase [EC:4.2.1.134] |
| 13 | ko00071 | Fatty acid degradation | ACAA1 | acetyl-CoA acyltransferase 1 [EC:2.3.1.16] |
| 14 | ko00280 | Valine, leucine and isoleucine degradation | AGXT2 | alanine-glyoxylate transaminase / (R)-3-amino-2-methylpropionate-pyruvate transaminase [EC:2.6.1.44 2.6.1.40] |
|  |  |  | ACAA1 | acetyl-CoA acyltransferase 1 [EC:2.3.1.16] |
| 15 | ko00592 | alpha-Linolenic acid metabolism | ACAA1 | acetyl-CoA acyltransferase 1 [EC:2.3.1.16] |
| 16 | ko04146 | Peroxisome | ACAA1 | acetyl-CoA acyltransferase 1 [EC:2.3.1.16] |
|  |  |  | HAO | (S)-2-hydroxy-acid oxidase [EC:1.1.3.15] |
| 17 | ko00073 | Cutin, suberine and wax biosynthesis | HHT1 | omega-hydroxypalmitate O-feruloyl transferase [EC:2.3.1.188] |
| 18 | ko00100 | Steroid biosynthesis | LIPA | lysosomal acid lipase/cholesteryl ester hydrolase [EC:3.1.1.13] |
| 19 | ko00190 | Oxidative phosphorylation | PMA1, PMA2 | H+-transporting ATPase [EC:7.1.2.1] |

continued table:

|  | Pathway | Pathway definition | KEGG gene name | KEGG description |
| --- | --- | --- | --- | --- |
| 20 | ko00195 | Photosynthesis | psbR | photosystem II 10kDa protein |
|  |  |  | petH | ferredoxin-NADP^+^ reductase [EC:1.18.1.2] |
| 21 | ko00196 | Photosynthesis-antenna proteins | LHCB2 | light-harvesting complex II chlorophyll a/b binding protein 2 |
| 22 | ko00220 | Arginine biosynthesis | argC | N-acetyl-gamma-glutamyl-phosphate reductase [EC:1.2.1.38] |
| 23 | ko00250 | Alanine, aspartate and glutamate metabolism | AGXT2 | alanine-glyoxylate transaminase / (R)-3-amino-2-methylpropionate-pyruvate transaminase [EC:2.6.1.44 2.6.1.40] |
| 24 | ko00260 | Glycine, serine and threonine metabolism | purA, ADSS | adenylosuccinate synthase [EC:6.3.4.4] |
|  |  |  | SRR | serine racemase [EC:5.1.1.18] |
|  |  |  | E4.3.1.19, ilvA, tdcB | threonine dehydratase [EC:4.3.1.19] |
| 25 | ko00270 | Cysteine and methionine metabolism | AGXT2 | alanine-glyoxylate transaminase / (R)-3-amino-2-methylpropionate-pyruvate transaminase [EC:2.6.1.44 2.6.1.40] |
| 26 | ko00290 | Valine, leucine and isoleucine biosynthesis | E4.3.1.19, ilvA, tdcB | threonine dehydratase [EC:4.3.1.19] |
| 27 | ko00310 | Lysine degradation | AASS | alpha-aminoadipic semialdehyde synthase [EC:1.5.1.8 1.5.1.9] |
| 28 | ko00400 | Phenylalanine, tyrosine and tryptophan biosynthesis | trpE | anthranilate synthase component I [EC:4.1.3.27] |
| 29 | ko00480 | Glutathione metabolism | pepN | aminopeptidase N [EC:3.4.11.2] |
| 30 | ko00500 | Starch and sucrose metabolism | E3.2.1.2 | beta-amylase [EC:3.2.1.2] |
|  |  |  | WAXY | granule-bound starch synthase [EC:2.4.1.242] |
| 31 | ko00564 | Glycerophospholipid metabolism | ETNK, EKI | ethanolamine kinase [EC:2.7.1.82] |
| 32 | ko00630 | Glyoxylate and dicarboxylate metabolism | HAO | (S)-2-hydroxy-acid oxidase [EC:1.1.3.15] |
|  |  |  | rbcS | ribulose-bisphosphate carboxylase small chain [EC:4.1.1.39] |

continued table:

|  | Pathway | Pathway definition | KEGG gene name | KEGG description |
| --- | --- | --- | --- | --- |
| 33 | ko00740 | Riboflavin metabolism | ACP5 | tartrate-resistant acid phosphatase type 5 [EC:3.1.3.2] |
| 34 | ko00760 | Nicotinate and nicotinamide metabolism | SDT1 | pyrimidine and pyridine-specific 5'-nucleotidase [EC:3.1.3.-] |
| 35 | ko00790 | Folate biosynthesis | FPGS | folylpolyglutamate synthase [EC:6.3.2.17] |
| 36 | ko00906 | Carotenoid biosynthesis | CYP707A | (+)-abscisic acid 8'-hydroxylase [EC:1.14.14.137] |
| 37 | ko00940 | Phenylpropanoid biosynthesis | E1.11.1.7 | peroxidase [EC:1.11.1.7] |
| 38 | ko03008 | Ribosome biogenesis in eukaryotes | orn, REX2, REXO2 | oligoribonuclease |
| 39 | ko03010 | Ribosome | RP-S13, rpsM | small subunit ribosomal protein S13 |
|  |  |  | RP-L31, rpmE | large subunit ribosomal protein L31 |
| 40 | ko03013 | RNA transport | EIF1, SUI1 | translation initiation factor 1 |
| 41 | ko03015 | mRNA surveillance pathway | PPP2R5 | serine/threonine-protein phosphatase 2A regulatory subunit B' |
| 42 | ko03020 | RNA polymerase | RPB3, POLR2C | DNA-directed RNA polymerase II subunit RPB3 |
| 43 | ko03022 | Basal transcription factors | TFIIH4, GTF2H4, TFB2 | transcription initiation factor TFIIH subunit 4 |
| 45 | ko03040 | Spliceosome | PRPF38B | pre-mRNA-splicing factor 38B |
|  |  |  | HSPA1s | heat shock 70kDa protein 1/2/6/8 |
| 46 | ko04141 | Protein processing in endoplasmic reticulum | RNF5 | E3 ubiquitin-protein ligase RNF5 [EC:2.3.2.27] |
|  |  |  | HSPA1s | heat shock 70kDa protein 1/2/6/8 |
| 47 | ko04144 | Endocytosis | DNM1_3 | dynamin 1/3 [EC:3.6.5.5] |
|  |  |  | IST1 | vacuolar protein sorting-associated protein IST1 |
|  |  |  | HSPA1s | heat shock 70kDa protein 1/2/6/8 |
| 48 | ko03050 | Proteasome | PSMD3, RPN3 | 26S proteasome regulatory subunit N3 |
| 49 | ko03430 | Mismatch repair | mutS2 | DNA mismatch repair protein MutS2 |

continued table:

|  | Pathway | Pathway definition | KEGG gene name | KEGG description |
| --- | --- | --- | --- | --- |
| 50 | ko03440 | Homologous recombination | RAD54L, RAD54 | DNA repair and recombination protein RAD54 and RAD54-like protein [EC:3.6.4.-] |
| 51 | ko04016 | MAPK signaling pathway - plan | MPK3 | mitogen-activated protein kinase 3 [EC:2.7.11.24] |
|  |  |  | PP2C | protein phosphatase 2C [EC:3.1.3.16] |
|  |  |  | FLS2 | LRR receptor-like serine/threonine-protein kinase FLS2 [EC:2.7.11.1] |
| 52 | ko04075 | Plant hormone signal transduction | PP2C | protein phosphatase 2C [EC:3.1.3.16] |
| 53 | ko04626 | Plant-pathogen interaction | MPK3 | mitogen-activated protein kinase 3 [EC:2.7.11.24] |
|  |  |  | FLS2 | LRR receptor-like serine/threonine-protein kinase FLS2 [EC:2.7.11.1] |
|  |  |  | CERK1 | chitin elicitor receptor kinase 1 |
|  |  |  | EDS1 | enhanced disease susceptibility 1 protein |
| 54 | ko04120 | Ubiquitin mediated proteolysis | SIAH1 | E3 ubiquitin-protein ligase SIAH1 [EC:2.3.2.27] |
|  |  |  | RCHY1, PIRH2 | RING finger and CHY zinc finger domain-containing protein 1 [EC:2.3.2.27] |
