# Supplementary material for: Physiological and transcriptomic analysis of cranberry (Vaccinium macrocarpon) in response to drought stress
Source: Front Plant Sci. 2026 May 7;17:1797317. doi: 10.3389/fpls.2026.1797317 (PMC13189740; doi:10.3389/fpls.2026.1797317)
Supplement: Supplementary Figure 1 — Venn diagram of common KEGG enriched pathways of differentially expressed genes in cranberry under different drought stress treatments [file Supplementaryfile1.docx]

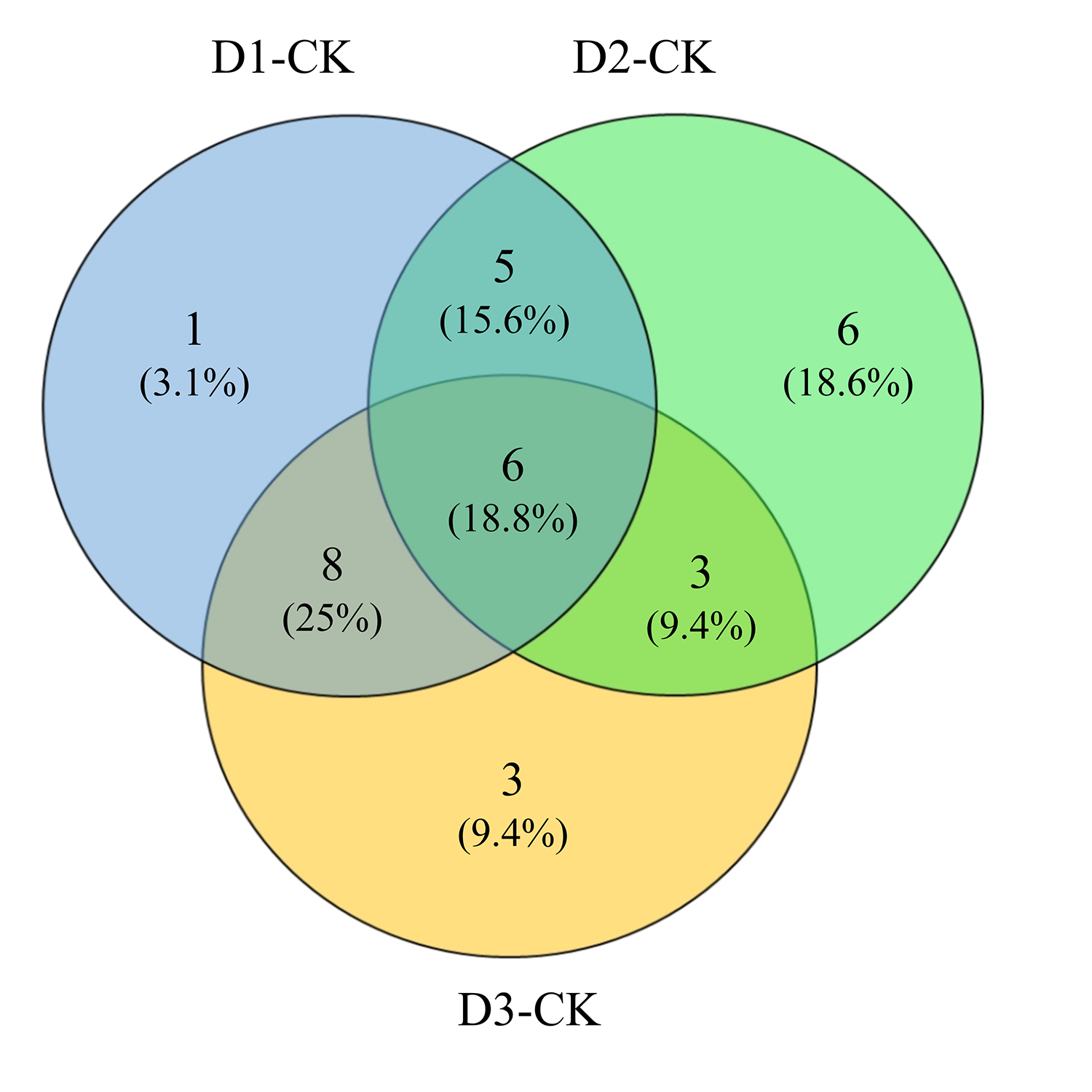


**Supplementary Figure S1 Venn diagram of common KEGG enriched pathways of differentially expressed genes in cranberry under different drought stress treatments**
