# Supplementary material for: Physiological and transcriptomic analysis of cranberry (Vaccinium macrocarpon) in response to drought stress
Source: Front Plant Sci. 2026 May 7;17:1797317. doi: 10.3389/fpls.2026.1797317 (PMC13189740; doi:10.3389/fpls.2026.1797317)
Supplement: Supplementary Figure 2 — Common differentially expressed genes of cranberry in response to KEGG pathway under different drought stress. [file Supplementaryfile2.docx]

**
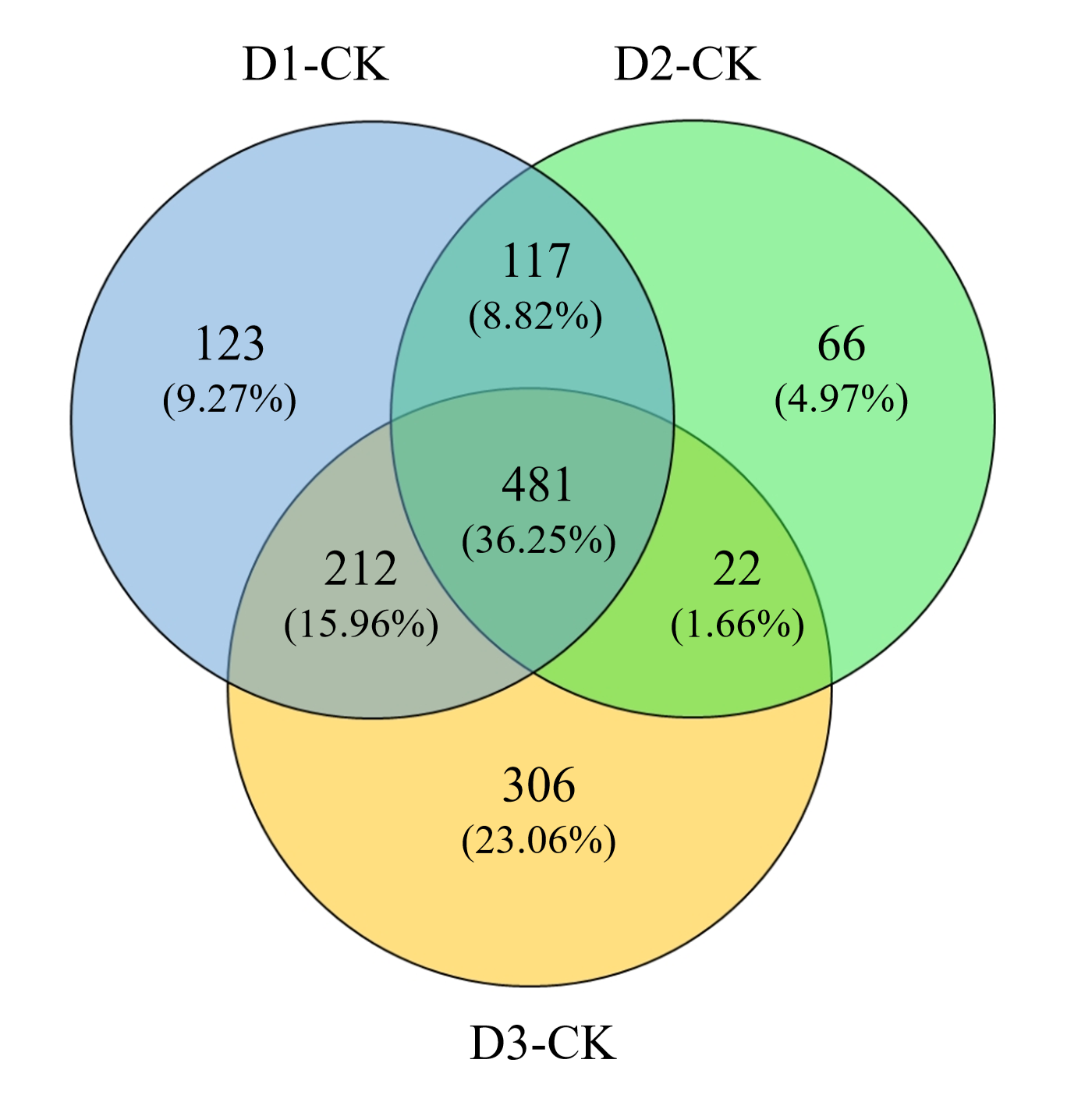
**

**Supplementary Figure S2 Common differentially expressed genes of cranberry in response to KEGG pathway under different drought stress**
